# Exosomal miR-155-5p derived from glioma stem-like cells promotes mesenchymal transition via targeting ACOT12

**DOI:** 10.1038/s41419-022-05097-w

**Published:** 2022-08-19

**Authors:** Zixu Bao, Ning Zhang, Wanxiang Niu, Maolin Mu, Xiaoming Zhang, Shanshan Hu, Chaoshi Niu

**Affiliations:** 1 Department of Neurosurgery, The First Affiliated Hospital of USTC, Division of Life Sciences and Medicine, University of Science and Technology of China, 230001 Hefei, Anhui People’s Republic of China; 2 Anhui Key Laboratory of Brain Function and Diseases, 230001 Hefei, Anhui People’s Republic of China; 3 Anhui Neurological Institute, 230001 Hefei, Anhui People’s Republic of China

**Keywords:** CNS cancer, Small RNAs

## Abstract

Tumor-associated exosomes play essential roles in intercellular communication and the foundation of cancer microenvironment in glioma. Many mRNAs, microRNAs (miRNAs) and proteins contained in tumor-associated exosomes can be transferred to recipient cells and contribute to the progression of tumor. Nevertheless, the cellular communication between malignant cells with different heterogeneities or characteristics and resultant tumor progression are still unclear in glioma. Here, we show that exosomes released from glioma stem-like cells (GSCs) contain a significant increasing level of miR-155-5p and could be horizontally transferred to surrounding glioma cells. High expression of miR-155-5p in plasma exosomes from patients was associated with glioma diagnosis and grading. Mechanically, we found that miR-155-5p markedly reduced the expression of acetyl-CoA thioesterase 12 (ACOT12), which played as a tumor suppressor in glioma. Furthermore, mesenchymal transition was significantly promoted in glioma cells treated with GSCs-derived exosomes. In conclusion, GSCs-derived exosomal miR-155-5p play a critical role in glioma progression and facilitating tumor aggressive growth by targeting ACOT12 and promoting mesenchymal transition. Exosomal miR-155-5p is also a potential predictive biomarker for glioma, which may provoke the development of novel diagnostic and therapeutic strategies against glioma.

## Introduction

Glioma is the most common primary malignant tumor of the central nervous system and has a high incidence rate ranging from 4.67–5.73 per 100,000 persons. Despite significant progress made in surgery, radiation and chemotherapy, the overall 5-year survival rate for patients with aggressive glioma remains <10% [1, 2]. Recent studies have indicated that this poor therapeutic outcome is attributed to glioma stem-like cells (GSCs), which are an aggressive population of tumor cells displaying a high capacity for self-renewal and therapeutic resistance [2, 3]. GSCs often enhance the invasiveness of tumor cells and contribute to rapid aggressive growth in brain [4, 5], but the molecular mechanisms are not fully understood.

Exosomes, which are small (30–100 nm) extracellular vesicles secreted from multiple types of cells, exist in almost all biological fluids [6]. Exosomes can participate in cell communication by transferring intracellular cargoes, including proteins and coding and noncoding RNAs, which are able to exert various biological functions [7]. Such intercellular communication between cells is essentially mediated by extracellular RNAs, including microRNAs (miRNAs), which are substantially enriched in exosomes [7, 8]. Furthermore, current evidence suggests that tumor cells actively secrete exosomes that participate in many processes in tumor progression, such as formation of the tumor microenvironment, escape from immunosurveillance and formation of a premetastatic niche [9–11]. Epithelial mesenchymal transition (EMT), which is a crucial step for metastasis in many solid tumors, is also promoted by tumor-associated exosomes. Hypoxic cancer cell-derived exosomes enrich miR-301a-3p, which facilitates the migration, invasion and EMT in pancreatic cancer [12]. Exosomal miR-92a-3p derived from hepatocellular cancer cells with high metastatic potentials also significantly upregulates EMT by targeting PTEN/Akt pathway [13]. With regard to glioma, GSCs critically participate in EMT, which is called mesenchymal transition in this paper because glioma is not of epithelial origin [14, 15]. Also, exosomes released from GSCs are involved in tumor growth, angiogenesis, and chemoresistance, which result in a poor prognosis [16–18]. Thus, more investigations are needed to clarify how GSCs-derived exosomes enhance the malignancy of tumor, and whether promotes mesenchymal transition in glioma.

In this study, we confirmed that GSCs-derived exosomes have an elevated level of miR-155-5p, which is also upregulated in plasma exosomes from individuals with glioma. Notably, miR-155-5p can repress the expression of ACOT12, which was shown to enhance the invasion and migration ability of glioma cells by facilitating EMT progression. Overall, we proved that exosomal miR-155-5p might be a novel diagnostic and therapeutic target for malignant glioma.

## Results

### Isolation and characterization of glioma stem-like cells and exosomes

**Fig. 1:**
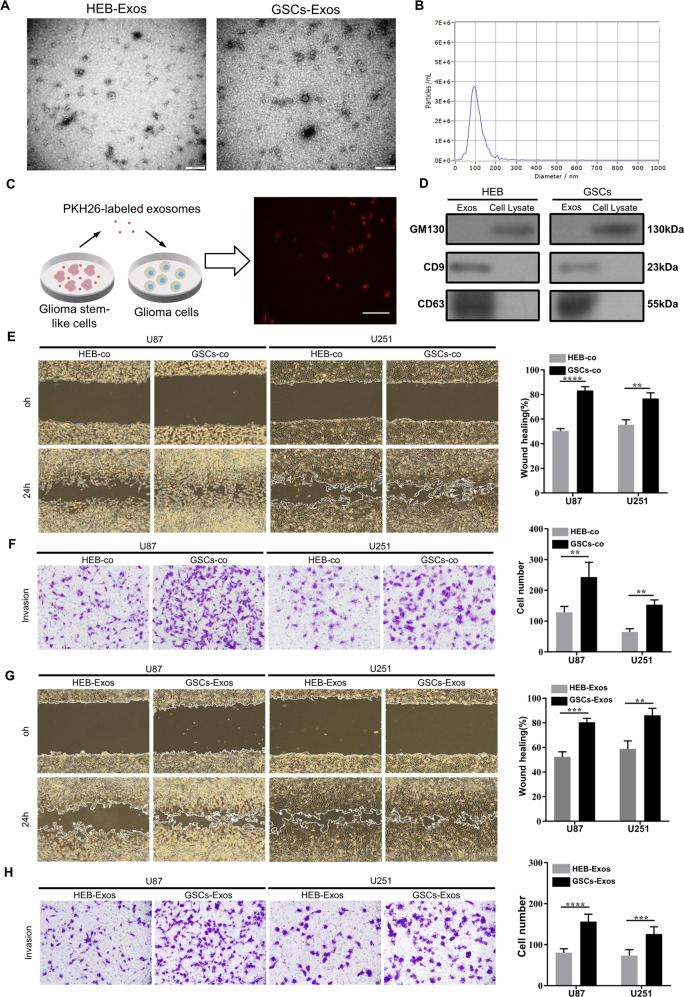
Identification of exosomes and the effect of GSCs-derived exosomes on the aggressiveness of glioma cells. **A** Respective transmission electron microscopy analysis of exosomes secreted by GSCs and HEB cells. Scale bar, 200 nm; **B** The particle size of isolated exosomes was measured by NTA; **C** Representative confocal fluorescence microscopy image showing the internalization of PKH26 (red)-labeled exosomes by glioma cells. Scale bar, 100 μm; **D** Exosome surface markers CD63 and CD9 and the negative control marker GM130 were detected by western blotting; **E** Wound healing assays in U87 and U251 cells after co-culture with GSCs; **F** Transwell assays in U87 and U251 cells after co-culture with GSCs. **G** Wound healing assays in U87 and U251 cells incubated with GSCs-derived exosomes or HEB-derived exosomes; **H** Transwell assays in U87 and U251 cells incubated with GSCs-derived exosomes or HEB-derived exosomes. Representative images are shown. Data were from three independent experiments and expressed as the mean ± SEM; **P < 0.01; ***P < 0.001; ****P < 0.0001 (Student’s *t*-test).

Glioma stem-like cells were isolated from U87 cells with neural sphere culture method and cultured in stem cell medium. The GSCs-specific markers CD133 and Nestin were stained positive by immunofluorescence in cultured spheres (Supplementary File 1A). Exosomes derived from GSCs and human normal glial cells (HEB cells) were extracted from the supernatant of the culture medium using ultracentrifugation. The morphological identifications and characteristic sizes of exosomes were determined by transmission electron microscopy (TEM) and nanoparticle tracking analysis (NTA) (Fig. 1A, B). To verify whether GSCs-derived exosomes can be taken up by glioma cells, GSCs-derived exosomes were labeled with the fluorescent dye PKH26 and then incubated with glioma cells. The presence of intracellular red fluorescence staining indicated that glioma cells could efficiently internalized the GSCs-derived exosomes (Fig. 1C). The exosome-specific protein markers CD63 and CD9 and the cellular negative control protein GM130 were detected by western blotting (Fig. 1D). These results indicate that GSCs can secrete exosomes that are efficiently internalized by recipient cells.

### GSCs induce the migration and invasion of glioma cells via exosomes

To explore the effects of GSCs on glioma progression and its mechanisms, we co-cultured glioma cells with GSCs or HEB cells. The migration and invasion of glioma cells were significantly increased, as detected by wound healing and Transwell assays (Fig. 1E, F and Supplementary File 1B). To determine whether exosomes alone can promote glioma progression, we then treated glioma cells with exosomes secreted by GSCs or HEB cells. Wound healing and Transwell assays further indicated that GSCs-derived exosomes significantly enhanced the invasiveness of recipient cells compared with HEB-derived exosomes. (Fig. 1G, H and Supplementary File 1C). Taken together, our results suggest that exosomes play a vital role in mediating the effect of GSCs on the migration and invasion of glioma.

### MiRNA expression profile of GSCs-derived exosomes and validation of elevated exosomal miR-155-5p

**Fig. 2:**
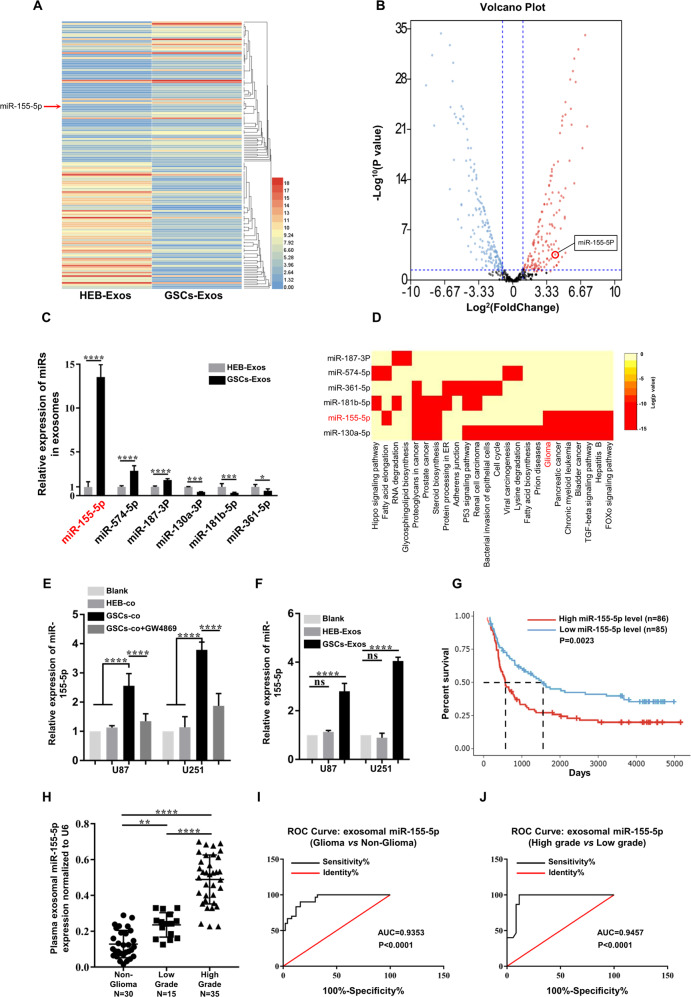
The expression of exosomal miR-155-5p in GSCs-derived exosomes and in the plasma of glioma patients. **A** Heatmap diagram of different expression levels of exosomal miRNAs between GSCs and HEB cells obtained using deep sequencing on the BGISEQ-500 platform; **B** Volcano plot of the differentially expressed miRNAs; **C** Different expression levels of exosomal miRNAs in GSCs-derived and HEB-derived exosomes were validated by RT-qPCR; **D** KEGG pathway analysis of exosomal miRNAs with remarkable differences; **E** The expression of miR-155-5p in glioma cells after co-culture with GSCs or GSCs treated with GW4869; **F** The effect of GSCs-derived exosomes on the expression level of miR-155-5p in glioma cells detected by RT-qPCR; **G** Kaplan–Meier plot and statistics for overall survival in glioma patients with high (*n* = 86) and low (*n* = 85) levels of miR-155-5p (from the TCGA), *P*-value = 0.0023; **H** Exosomal miR-155-5p in the plasma of patients with different grades of glioma determined by RT-qPCR; **I** ROC curves showing the ability of exosomal miR-155-5p in plasma to discriminate glioma patients, *P*-value < 0.0001; **J** ROC curves showing the ability of exosomal miR-155-5p in plasma to distinguish high-grade glioma and low-grade glioma; *P*-value < 0.001. Data represent as the mean ± SEM from three independent experiments; *P < 0.05; **P < 0.01; ***P < 0.001; ****P < 0.0001 (Student’s *t*-test).

Many researchers have indicated that GSCs-derived exosomes participate in angiogenesis, tumor microenvironment and chemoresistance by transferring miRNAs, which contribute to the development and progression of malignant glioma [17, 19, 20]. To assess the changes in the expression of GSCs-derived exosomal miRNAs, we conducted deep sequencing to investigate the miRNA expression profiles of exosomes secreted by GSCs and HEB cells. All produced reads were compared with the known human miRNAs in miRbase, and 1155 miRNAs were identified. The highly represented miRNAs with the most significant (fold-change >3, *P* < 0.05) difference are shown in Fig. 2A. A total of 156 miRNAs were identified as significantly dysregulated in GSCs-derived exosomes compared with HEB-derived exosomes; 83 were upregulated, and 73 were downregulated (Fig. 2B). To validate the deep sequencing results, we randomly selected to detect the exosomal levels of miR-155-5p, miR-574-5p, miR-187-3p, miR-130a-3p, miR-181b-5p, and miR-361-5p by RT-qPCR (Fig. 2C). Among the identified miRNAs, miR-155-5p exhibited the greatest changes; this miRNA has also been demonstrated to promote glioma development and progression [21]. By searching the Kyoto Encyclopedia of Genes and Genomes (KEGG) pathway database, we found that miR-155-5p was significantly enriched in the glioma downstream signaling pathway (Fig. 2D). Additionally, the level of miR-155-5p in glioma cells was obviously increased after co-culture with GSCs and was reduced by the exosome secretion inhibitor GW4869 (Fig. 2E). A similar phenomenon was observed in glioma cells treated with GSCs-derived exosomes (Fig. 2F). Taken together, these results indicate that GSCs-derived exosomes containing abundant miR-155-5p can be transferred to recipient glioma cells and elevate miR-155-5p levels. In addition, Kaplan–Meier analysis of the overall survival of glioma patients indicated that patients with high expression levels of miR-155-5p were more likely to have a poor prognosis (Fig. 2G). To explore the correlation between plasma exosomal miR-155-5p and glioma, we subsequently assayed the levels of plasma exosomal miR-155-5p from 50 patients with glioma (Table 1) and 30 patients with nonglioma diseases. The results indicated that the expression of miR-155-5p from the plasma exosomes was significantly elevated in both the high- and low-grade glioma groups (Fig. 2H). To evaluate the diagnostic value of plasma exosomal miR-155-5p, receiver operating characteristic (ROC) curves were used to discriminate glioma/nonglioma patients or patients with gliomas of different grades. The area under the curve (AUC) for plasma exosomal miR-155-5p in the glioma/nonglioma groups was 0.9353 (95% CI: 0.8864 to 0.9843), which suggested that the level of plasma exosomal miR-155-5p could be an excellent diagnostic marker to distinguish glioma from nonglioma diseases (Fig. 2I). Moreover, the AUC in the high/low-grade glioma group was 0.9457 (95% CI: 0.885 to 1.006), which indicated that plasma exosomal miR-155-5p can be used to separate high-grade and low-grade glioma patients (Fig. 2J). Therefore, plasma exosomal miR-155-5p exhibits great potential as a novel fluid biopsy indicator to diagnose and grade glioma.

**Table 1:** Blood samples information of 50 glioma patients.

Variable	Samples (*n* = 50)
Gender	
Male	18
Female	32
Ages	
≤45	17
>45	33
WHO grade	
I	5
II	10
III	14
IV	21
Location	
Frontal	25
Parietal	4
Occipital	6
Temporal	15
Histology	
Oligodendroglioma	3
Astrocytoma	26
Glioblastoma	21

### The effect of GSCs-derived exosomes on the invasion and migration of glioma cells is mediated by miR-155-5p

**Fig. 3:**
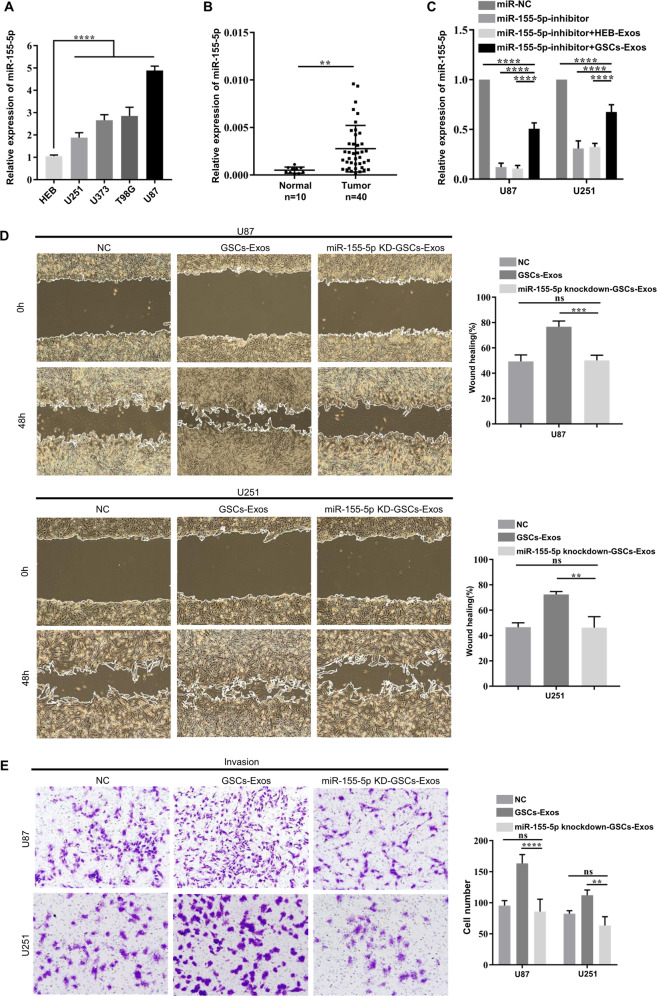
The effect of GSCs-derived exosomes on migration and invasion is mediated by miR-155-5p. **A** RT-qPCR analysis of the relative expression of miR-155-5p in HEB cells and 4 glioma cell lines; **B** Relative expression of miR-155-5p in 10 normal brain tissues and 40 glioma tissues; **C** Relative expression of miR-155-5p in U87 and U251 cells treated with miR-155-5p inhibitor and exosomes secreted by GSCs or HEB cells; **D** The migration of glioma cells treated with miR-155-5p inhibitor and GSCs-derived or HEB-derived exosomes was determined by wound healing assays; **E** The invasion of glioma cells treated with miR-155-5p inhibitor and exosomes secreted by GSCs or HEB was assessed with Matrigel-coated Transwell assays. Representative images are shown. Data represent as the mean ± SEM (repetition = 3); ns not statistically significant; **P < 0.01; ***P < 0.001; ****P < 0.0001 (Student’s *t*-test).

To select suitable glioma cell lines for further experiments, we detected the expression of miR-155-5p in four glioma cell lines and verified that U87 and U251 cells were suitable for subsequent analyses (Fig. 3A). We also confirmed the upregulation of miR-155-5p in glioma tissues compared to normal controls (Fig. 3B). We further elucidated the effect of GSCs-derived exosomes on the expression level of miR-155-5p in recipient cells. The level of miR-155-5p was significantly decreased in U87 and U251 cells transfected with a miR-155-5p-specific inhibitor. However, compared with HEB-derived exosomes, GSCs-derived exosomes were able to partially restore the content of miR-155-5p in recipient glioma cells (Fig. 3C). It has been reported that miR-155-5p can promote the migration and invasion of cancer cells in a variety of tumors, including glioma [21–23], which was confirmed by our results in Supplementary File 1E and 2A, B. We then intended to explore whether the promoting effect of GSCs-derived exosomes on invasiveness is mediated by exosomal miR-155-5p. We established the miR-155-5p stably knockdown GSCs by lentivirus and detected the level of exosomal miR-155-5p from its culture medium (Supplementary File 2C, D). The result showed that depletion of miR-155-5p in GSCs could also attenuate the level of exosomal miR-155-5p. As depicted in Fig. 3D, E, GSCs-derived exosomes containing low level of miR-155-5p had no promoting effect on invasiveness, while GSCs-derived exosomes with high level of miR-155-5p obviously contributed to invasion and migration in glioma cells. Taken together, these results demonstrate that the effect of GSCs-derived exosomes on migration and invasion is predominantly mediated by the transfer of exosomal miR-155-5p.

### MiR-155-5p facilitates the migration and invasion of glioma cells in vivo

**Fig. 4:**
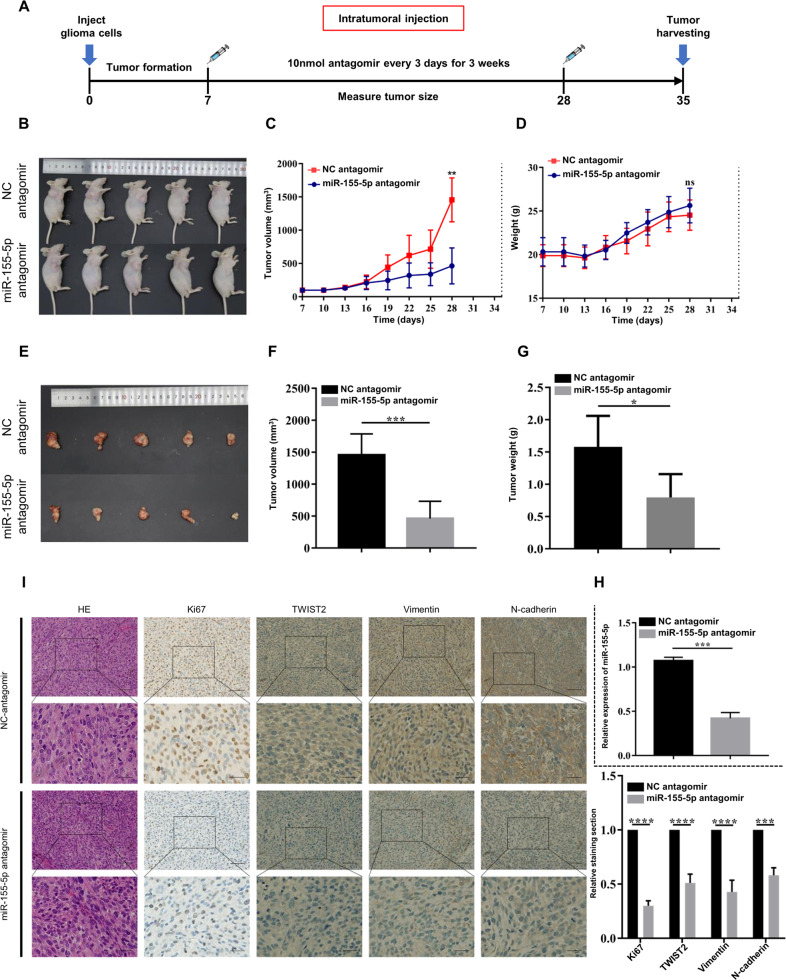
Specific inhibition of miR-155-5p can suppress glioma progression in vivo. **A** Illustration of the xenograft experimental process. Divergent arrows indicate the different stages; **B** Establishment of glioma xenografts in nude mice (2 groups, mice=5/group); **C**, **D** Tumor volume and mice weight were measured every 3 days; **E** Images of dissected tumors; **F**, **G** Volumes and weights of the dissected tumors treated with miR-155-5p antagomir or normal control; **H** Relative expression of miR-155-5p in xenograft tissues was measured by RT-Qpcr; **I** Relative expression of Ki67, TWIST2, vimentin and N-cadherin in xenograft tissues treated with miR-155-5p antagomir or NC was revealed using IHC staining. Representative images are shown. Scale bar, up: 100 μm; down: 50 μm. Data were expressed as the mean ± SEM (repetition = 3); ns not statistically significant; *P < 0.05; ***P < 0.001; ****P < 0.0001 (Student’s *t*-test).

Subsequently, we established a xenograft tumor model in nude mice to clarify the effect of miR-155-5p on the migration and invasion of glioma in vivo. The subsequent workflow of the in vivo experiments is illustrated in Fig. 4A. When the tumor models were well established, we performed intratumoral multiple-point injection with miR-155-5p antagomir, which can specifically restrain miR-155-5p in xenograft model and NC antagomir every 3 days 7 times as described above. The monitoring results showed that interjection with miR-155-5p antagomir could significantly inhibit the growth of tumors (Fig. 4B, C). Besides, no obvious difference in body weight was observed between the two groups, which can be used to exclude the detrimental side effects of antagomir (Fig. 4D). After 28 days, mice were sacrificed to obtain tumor tissues (Fig. 4E). The volume and weight of tumor tissues were measured, which also suggested that miR-155-5p antagomir can suppress the development of tumors (Fig. 4F, G). Downregulation efficiency of miR-155-5p in xenograft tissues was revealed by RT-qPCR (Fig. 4H). Furthermore, the IHC results demonstrated that the expression levels of malignant markers (Ki67) and mesenchymal markers (TWIST2, vimentin and N-cad) were apparently reduced after treatment with miR-155-5p antagomir (Fig. 4I). These findings suggest that miR-155-5p promotes the progression of glioma and specific inhibition of miR-155-5p restrains the migration and invasion of glioma cells in vivo.

### Exosomal miR-155-5p enhances the migration and invasion of glioma cells by targeting ACOT12

**Fig. 5:**
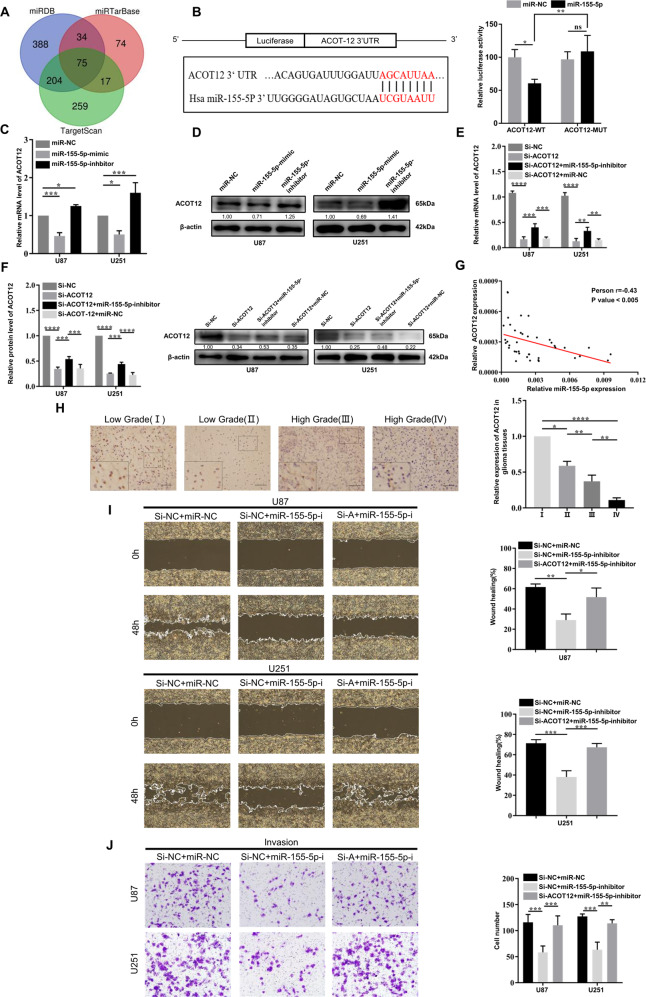
ACOT12 is the downstream target of miR-155-5p and inhibits glioma development. **A** Prediction of downstream target genes of miR-155-5p via four online databases (miRDB, TargetScan, miRTarBase); **B** Sequence alignment showing the potential binding sites between ACOT12 mRNA and miR-155-5p; Luciferase reporter activity of WT or MUT ACOT12 in 293 T cells co-transfected with miR-155-5p mimic; **C**, **D** Relative mRNA and protein levels of ACOT12 in glioma cells transfected with miR-155-5p inhibitor of mimic; **E**, **F** Relative expression level of ACOT12 in glioma cells transfected with si-ACOT12 alone or co-transfected with si-ACOT12 and miR-155-5p inhibitor detected by RT-qPCR and western blotting; **G** The Pearson correlation between miR-155-5p level and ACOT12 level was measured in glioma tissues. The ΔCt values (normalized to GAPDH) were subjected to Pearson correlation analysis (*R* = −0.43, *P* < 0.005); **H** Relative expression level of ACOT12 in glioma sections of different grades detected by IHC; **I**, **J** Migration and invasion of glioma cells transfected with miR-155-5p inhibitor or co-transfected with ACOT12 siRNA and miR-155-5p inhibitor, as detected by wound healing assays and Matrigel-coated Transwell assays. Representative images are shown. Data represent as the mean ± SEM from three independent experiments. *P < 0.05; **P < 0.01; ***P < 0.001; ****P < 0.0001 (Student’s *t-*test).

To elucidate the mechanisms by which exosomal miR-155-5p participates in the migration and invasion of glioma cells, we performed comprehensive bioinformatic analysis using three online databases to predict potential targets of miR-155-5p (Fig. 5A). A total of 75 mRNAs were predicted to be targeted by miR-155-5p, potentially including acetyl-CoA thioesterase 12 (ACOT12), which was first reported as a tumor suppressor by regulating EMT in HCC [24]. Thus, we hypothesized that miR-155-5p contributes to migration and invasion in glioma by targeting ACOT12, which in turn promotes mesenchymal transition.

To verify this conjecture, dual-luciferase assays were performed to validate the interaction between miR-155-5p and ACOT12. The results showed that the luciferase activity of ACOT12-WT was obviously decreased after co-transfection of miR-155-5p mimics. However, no significant difference was observed for ACOT12-MUT co-transfected with miR-155-5p mimics (Fig. 5B). The effect of miR-155-5p on the expression of ACOT12 was determined by RT-qPCR and western blotting. The results demonstrated that the level of ACOT12 was obviously elevated after inhibition of miR-155-5p, whereas overexpression of miR-155-5p significantly reduced the expression of ACOT12 (Fig. 5C, D). We then investigated the role of ACOT12 in glioma progression. For gain-of-function and loss-of-function experiments, a specific siRNA was used to inhibit the expression of ACOT12. The miR-155-5p inhibitor was able to restore the expression of ACOT12, which was validated by RT-qPCR and western blotting (Fig. 5E, F). Correlation analysis also indicated a negative relationship between expression of ACOT12 and miR-155-5p in glioma tissues (Fig. 5G). Subsequently, the expression level of ACOT12 was detected by RT-qPCR in four glioma cell lines and HEB cells, and the results indicated that ACOT12 was significantly downregulated in glioma cells (Supplementary File 3A). We further confirmed the downregulation of ACOT12 in glioma tissues compared to normal controls (Supplementary File 3B). Remarkably, we also observed significant inverse correlations between ACOT12 expression and glioma grade by IHC analysis of clinical glioma paraffin sections. The results revealed that the expression of ACOT12 was significantly decreased with increasing glioma grade (Fig. 5H). The results of wound healing and Transwell assays indicated that specific inhibition of ACOT12 significantly enhanced the migration and invasion capabilities of glioma cells in vitro (Supplementary File 3C, D). Moreover, depletion of ACOT12 partially rescued the suppressive effect of miR-155-5p inhibition on migration and invasion in glioma cells (Fig. 5I, J and Supplementary File 3E). Taken together, these findings demonstrate that ACOT12 is the direct target gene of miR-155-5p and may act as a tumor suppressor in glioma.

### GSCs-derived exosomal miR-155-5p promotes glioma development by inducing mesenchymal transition

**Fig. 6:**
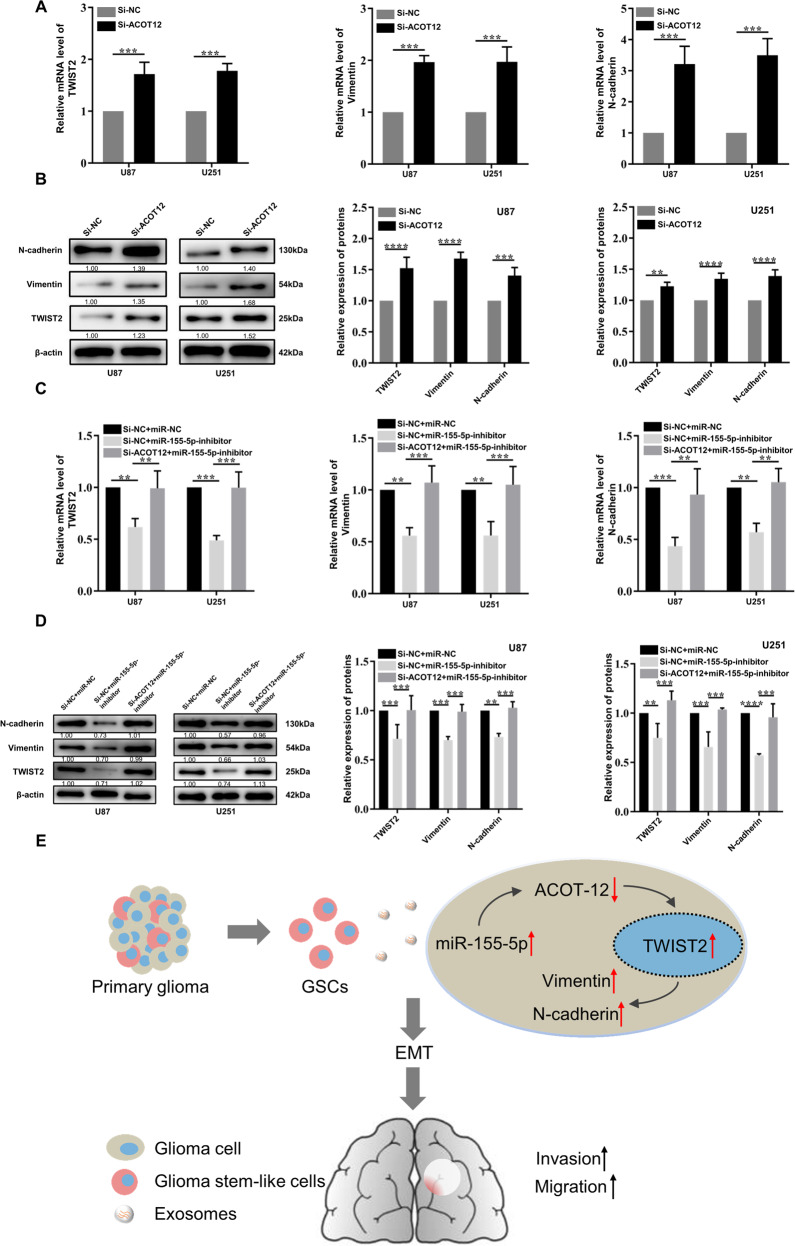
MiR-155-5p promotes mesenchymal transition by targeting ACOT12. **A**, **B** Relative expression levels of mesenchymal transition-related markers (TWIST2, vimentin and N-cadherin) in glioma cells transfected with si-ACOT12 determined by RT-qPCR and western blotting; **C**, **D** Relative mRNA and protein levels of mesenchymal transition-related markers in glioma cells transfected with miR-155-5p inhibitor alone or co-transfected with si-ACOT12 and miR-155-5p inhibitor; **E** Schematic diagram of the hypothetical mechanism of the GSCs-derived exosomal miR-155-5p enhances aggressiveness by targeting ACOT12 and promoting mesenchymal transition in glioma. Data represent as the mean ± SEM (repetition = 3). *P < 0.05; **P < 0.01; ***P < 0.001; ****P < 0.0001 (Student’s *t*-test).

Recently, ACOT12 has been reported to promote HCC metastasis by regulating TWIST2, which is the key transcription factor in EMT [24]. To explore whether the relationship between ACOT12 and EMT is also involved in the progression of glioma, we validated the effect of modulating ACOT12 expression on the expression levels of TWIST2 and other EMT markers, including vimentin and N-cadherin, by RT-qPCR and western blotting. The results showed that the expression of TWIST2 and EMT markers was significantly increased after knockdown of ACOT12 with siRNA, which indicated that depletion of ACOT12 was able to enhance mesenchymal transition in glioma cells (Fig. 6A, B). Furthermore, due to the targeting relationship between miR-155-5p and ACOT12, we investigated whether knockdown of ACOT12 could rescue the reducing effect of miR-155-5p inhibition on mesenchymal transition. The results showed that both TWIST2 and EMT markers were upregulated after transfection of ACOT12 siRNA versus the control (Fig. 6C, D). Since GSCs and GSCs-derived exosomes facilitate the migration and invasion of glioma cells, we further explored whether GSCs and GSCs-derived exosomes regulate mesenchymal transition. The results indicated that both co-culture with GSCs and treatment with GSCs-derived exosomes upregulated the expression of TWIST2 and EMT markers, indicating the promotion of mesenchymal transition (Supplementary File 4A–D). Additionally, overexpression of miR-155-5p contributed to the expression of TWIST2 and EMT markers. In contrast, depletion of miR-155-5p by a specific inhibitor reduced the expression of relevant markers, which could be restored after treatment with GSCs-derived exosomes (Supplementary File 5A–D). Taken together, our findings suggest that ACOT12 can serve as a tumor suppressor by regulating TWIST2 and mesenchymal transition in glioma. More importantly, we demonstrate that exosomal miR-155-5p derived from GSCs is able to target ACOT12 and accelerate mesenchymal transition, which in turn contributes to the migration and invasion of glioma cells (Fig. 6E).

## Discussion

Glioma is the most common type of primary malignant tumor of the central nervous system and the prognosis of patients with aggressive glioma remains extremely dismal [1, 2]. The invasiveness is the most common reason that residual tumor cells remain and local recurrence occurs [4, 25, 26]. Increasing evidence has demonstrated that glioma stem-like cells are strongly correlated with the immune suppression, tumor microenvironment and therapeutic resistance of glioblastoma [27]. Several researches indicated that GSCs targeted therapy could be the efficient strategy to prevent temozolomide resistance in glioblastoma treatment [28].

Exosome mediate cellular communication and exert diverse biological functions through the horizontal transfer of various cargos to recipient cells [6]. Recently, numerous studies have demonstrated that malignant cell-derived exosomes mediate tumor progression and aggressiveness via the transfer of noncoding RNAs, including microRNAs, long noncoding RNAs (lncRNAs) and circular RNAs (circRNAs) [7, 29]. For instance, cancer cell-derived exosomes induce the formation of premetastatic niches and distant metastasis in breast and colorectal cancer by transferring miR-21 and miR-25-3p, respectively [10, 30]. The exosomal transfer of lncRNA ZSAF1 enhances cancer cell proliferation and migration in gastric cancer [31]. CircRNA SATB2 participates in lung cancer progression by regulating miR-326/fascin homolog 1 and the actin-bundling protein 1 (FSCN1) loop and is highly expressed in serum exosomes [32]. Similarly, GSCs-derived exosomes participate in the tumor microenvironment by transferring miRNAs and lncRNAs, and this process promotes angiogenesis and chemoresistance in glioblastoma [17, 18]. Similar to these studies, in this paper, we explored the miRNA expression profiles of exosomes derived from GSCs and normal glial cells using deep sequencing. Based on the results, we found that GSCs-derived exosomes exhibited a higher expression level of miR-155-5p. Several studies have demonstrated that miR-155-5p promotes cancer progression in multiple tumors, including hepatocarcinoma, squamous cell carcinoma and colon cancer [22, 33, 34]. With regard to glioma, a recent study reported that miR-155-5p enhances the migration and invasion capability of glioma by directly targeting PCDH9 and activating the Wnt/β-catenin signaling pathway [21]. Besides, EMT has been proven to promote the development of glioma by facilitating cell invasion and migration [35–37]. Therefore, it is necessary to elucidate the relationship between exosomal miR-155-5p and mesenchymal transition in glioma progression and to identify the potential mechanism. In this work, we proved that overexpression of miR-155-5p facilitates the invasiveness of glioma cells and that specific inhibition of miR-155-5p is able to prevent glioma development in xenograft models. MiR-155-5p overexpression results in the upregulation of EMT markers, including TWIST2, vimentin and N-cadherin, suggesting that miR-155-5p promotes mesenchymal transition in glioma cells. Next, we found that GSCs-derived exosomes restored the malignant phenotype of glioma cells transfected with miR-155-5p inhibitor. We also identified that exosomes secreted from GSCs was transferred to recipient glioma cells to elevate the expression of miR-155-5p and promote mesenchymal transition, while the exosomes secretion inhibitor GW4869 significantly prevented this phenomenon, which proved that miR-155-5p was transferred by exosomes. Collectively, we demonstrated that GSCs-derived exosomes contribute to the invasiveness and aggressiveness of glioma by transmitting miR-155-5p to recipient glioma cells and inducing mesenchymal transition.

Previous studies have identified several downstream targets of miR-155-5p that function as tumor suppressors in glioma progression, including FOXO3a, MXI1, and HBP1 [38–40]. In this paper, we used three online databases to predict potential targets of miR-155-5p. Surprisingly, acetyl-CoA thioesterase 12 (ACOT12) was observed at the intersection of gene subsets and might be negatively correlated with miR-155-5p. The results of the dual-luciferase assay revealed that miR-155-5p directly targets ACOT12, and overexpression/downregulation of miR-155-5p also repressed/promoted ACOT12 expression. The main function of ACOT12 is as the key mediator for alteration of acetyl-CoA, which serves as a critical intermediator of carbon sources and participates in the production of ATP [41]. Current studies indicate that elevated levels of acetyl-CoA produced by active acetate metabolism are associated with the growth and metastasis of hepatocellular carcinoma (HCC), glioblastoma, breast cancer and prostate cancer [42–44]. However, the roles of ACOT12 in malignant glioma progression remain unknown. Interestingly, the latest report demonstrates that ACOT12 plays an inhibitory role in the metastasis of hepatocellular carcinoma and its expression is significantly decreased in highly metastatic HCC tissues. Downregulation of ACOT12 has also been suggested to significantly promote HCC distant metastasis via epigenetic induction of TWIST2, which contributes to the occurrence of EMT [24]. Thus, we hypothesized that ACOT12 might serve as a tumor suppressor in glioma progression. To explore the role of ACOT12 in glioma, we performed RT-qPCR and IHC experiments to reveal the expression of ACOT12 was significantly decreased in glioma tissues. In addition, a series of gain/loss-of-function experiments revealed that inhibition of ACOT12 could promote the migration and invasion of glioma cells by inducing mesenchymal transition. Downregulation of miR-155-5p attenuated the promoting effect of ACOT12 siRNA on the aggressiveness and mesenchymal transition of glioma cells. Collectively, our results indicate that GSCs-derived exosomal miR-155-5p is likely to regulate mesenchymal transition and enhance the aggressiveness of glioma by directly targeting ACOT12.

Recently, liquid biopsy has become a novel noninvasive strategy for the diagnosis of many diseases, including cancers; this process involves analyzing specific elements contained in body fluids, including blood, serum and cerebrospinal fluid (CSF). Through the analysis of circulating tumor cells, proteins, mRNA, miRNA and cell-free DNA, liquid biopsy can be applied to diagnose and monitor the progression of multiple cancers [45, 46]. Owing to their wide distribution in body fluids, extracellular vesicles, including exosomes, have attracted much attention in the diagnosis and treatment of glioma [47]. For example, LGALS9, which is a unique protein contained in exosomes from the CSF of glioblastoma patients, contributes to the inhibition of antitumor immunity and could be an effective indicator of glioblastoma and chemoresistance [48]. Aberrant expression of exosomal miR-21, miR-222, and miR-124-3p in the serum are closely related to tumor grade and a poor prognosis in patients with glioma [49, 50]. In our study, the level of exosomal miR-155-5p in plasma was determined by RT-qPCR and was found to be higher in glioma patients than in nonglioma patients. Furthermore, our results demonstrated that elevated expression of miR-155-5p in plasma from glioma patients is positively associated with tumor grade, suggesting that exosomal miR-155-5p could be a novel diagnostic biomarker for aggressive glioma.

In summary, we demonstrate that GSCs-derived exosomal miR-155-5p plays a critical role in enhancing the aggressiveness of glioma cells by targeting ACOT12 and promoting mesenchymal transition. Exosomes secreted by GSCs with elevated levels of miR-155-5p could be potential therapeutic entities for glioma. Moreover, exosomal miR-155-5p in plasma could serve as a novel noninvasive biomarker for the glioma diagnosis and tumor grading. Overall, this study may provide new insight into the mechanism of glioma progression and suggest a potential strategy for the diagnosis and treatment of glioma.

## Materials and methods

### Blood samples and human tissues

All clinical samples, including blood samples, human tissues and histological sections, were collected from the Department of Neurosurgery of the First Affiliated Hospital of the University of Science and Technology (USTC) between December 2019 and June 2020 with the approval of the Human Research Ethics Committee of the hospital. All the patients were informed and signed relevant consents. Blood samples from 50 patients with glioma and 30 patients with nonglioma diseases (Table 1). Briefly, blood samples (10 mL) were collected from each patient into vacuum tubes with ethylenediaminetetraacetic acid (EDTA) and centrifuged at 3000 × *g* for 10 min to isolate plasma. After isolation, the plasma was spun at 12,000 × *g* for 30 min to remove cell debris. The purified plasma (4–5 mL) was passed through a 0.22-µm filter (Millipore) and then stored in a sterile tube at −80 °C. All tumor tissues were obtained from 40 glioma patients (Table 2), and normal brain tissues were collected from 10 patients undergoing brain tissue resection due to craniocerebral injury.

**Table 2:** Tumor tissues information of 40 glioma patients.

Variable	Samples (*n* = 40)
Gender	
Male	17
Female	23
Ages	
≤45	9
>45	31
WHO grade	
I	2
II	7
III	11
IV	20
Location	
Frontal	25
Parietal	1
Occipital	4
Temporal	10
Histology	
Oligodendroglioma	1
Astrocytoma	21
Glioblastoma	18

### Cell culture

Human glioma cell lines, U251, U87, U373 and T98G, and a human normal glial cell line (HEB) were obtained from Shanghai Institute of Biochemistry and Cell Biology, Chinese Academy of Sciences (Shanghai, China) and maintained in our laboratory. All cell lines were authenticated by STR profiling and tested for mycoplasma contamination. All cell lines were cultured in DMEM (HyClone) containing 10% FBS (Clark Bioscience) and stored in a humidified incubator at 37 °C with 5% CO2. HEB cells were cultured in DMEM containing 10% exosome-depleted FBS (SBI, EXO-FBS-50A-1) so that the collected medium only contained HEB-derived exosomes. All cells used in experiments were performed with mycoplasma test.

### Glioma stem-like cells isolation and characterization

Glioma stem-like cells were isolated by neural sphere culture method as described before [51]. Briefly, U87 cells in logarithmic growth phase were collected in an aseptic centrifuge tube containing serum-free medium. Then, the separated U87 cells were resuspended in serum-free neural stem cell medium prepared with DMEM/F12 (Gibco) plus 20 ng/mL EGF (PeproTech), 20 ng/mL bFGF (PeproTech), and B27 supplements (Gibco) for 2 weeks. The resultant spheroids were defined as glioma stem-like cells. Fresh neural stem cell medium was added to the GSCs every 2 days. Immunofluorescence assays were performed to identify GSCs markers, including CD133 and Nestin.

### Exosome isolation, characterization, and RNA extraction

Exosomes were isolated as described by Ying et al. [52]. Briefly, GSCs culture medium and HEB cell culture medium with exosome-free serum were centrifuged at 1000 × *g* for 10 min and then filtered with a 0.22-µm filter (Millipore) to remove dead cells and debris. The supernatant was subjected to ultracentrifugation at 100,000 × *g* for 4–6 h at 4 °C and then washed with PBS at 100,000 × *g* for 20 min. The final pellet containing exosomes was resuspended in PBS. Western blotting was performed to identify exosome surface markers (CD63 and CD9) and the negative control (GM130). The morphology of exosomes was characterized by transmission electron microscopy (TEM) (Hitachi H-7650) and nanoparticle tracking analysis (NTA) (ZetaView PMX 110). Finally, the exosomes were labeled with PKH26 fluorescent dye using the PKH26 fluorescent cell linker kit (Sigma, PKH26GL) to monitor the transfer of GSCs-derived exosomes. Plasma exosomes and exosomal total RNA were extracted with an Exoeasy Max Kit (QIAGEN 77164) according to the manufacturer’s instructions.

### MiRNA library construction and deep sequencing

Total RNA extracted from exosomes was used for miRNA library preparation and sequencing. Library preparation and sequencing were performed at the Beijing Genomics Institution (BGI). Briefly, the concentration and purity of total exosomal RNA were identified using an Agilent 2100. Total exosomal RNA samples were fractionated, and small RNAs ranging from 18 to 30 nucleotides were used to construct the library. Small RNAs were reverse-transcribed and amplified by PCR. Subsequently, the PCR products were sequenced using the BGISEQ-500 platform.

### Cell transfection

Small-interfering RNAs (SiRNAs), miRNA mimics and inhibitors were obtained from RiboBio and transfected into recipient cells with Lipofectamine 3000 (Invitrogen) according to the manufacturer’s protocol. After 6 h, the culture medium was replaced with fresh medium. After 48 h, the transfection efficiencies were detected by RT-qPCR or western blotting, and the cells were used for subsequent experiments.

### Wound healing assay

Co-cultured or transfected U87 and U251 cells were rinsed with PBS 2 times when they reached 80% confluence in six-well plates. A sterile 200-μL pipette tip was used to create wounds, and the cells were cultured in medium containing 2% FBS for 48 h before the calculation of wound healing rates. To detect the effect of exosomes on wound healing, the cells were incubated with exosomes for 48 h after the formation of scratches.

The relative wound healing rates were detected by CellSense Standard Software (Olympus) to measure cell migration ability.

### Invasion and migration assay

Transwell assay inserts (Corning) were used as upper chambers to determine the invasive and migratory ability of glioma cells. To detect invasion, the upper chambers were coated with diluted Matrigel (BD) for 12 h at 37 °C. The bottom chambers were filled with 700 μL medium containing 10% FBS as a chemoattractant. Treated glioma cells (transfected, co-cultured or incubated) (1 × 10^4^ cells) were resuspended in 100 μL serum-free medium after serum starvation for 24 h and then seeded into the upper chamber. After incubation for 24 h (migration assay) or 48 h (invasion assay), cells that moved to the lower side of inserts were fixed with 4% paraformaldehyde for 20 min and stained with crystal violet solution for 15 min. Stained cells were imaged and counted with an inverted microscope (Olympus). Cell numbers from five random fields of each group were used for statistical analysis to measure invasion and migration.

### Co-culture assay

U87 and U251 cells were co-cultured with GSCs or HEB cells at a ratio of 1:1 for 24 h by using a 0.4-μm polycarbonate filter Transwell plate (Corning, 3450). GSCs or HEB cells were placed in the upper chamber, and glioma cells were seeded in the lower chamber. Since only exosomes were allowed to pass through the 0.4-μm polycarbonate filter, the effect of upper cell-derived exosomes on the lower cells was examined. The upper cells were treated with the exosome secretion inhibitor GW4869 (Sigma, D1692) as described previously [52] and then co-cultured with glioma cells as the other experimental group.

### Total RNA extraction and real-time quantitative PCR

Total RNA was extracted using TRIzol Reagent (Invitrogen) according to the manufacturer’s instructions. The concentration and purity of total RNA were determined by a NanoDrop ND-3300 (Thermo). Total RNA (500 ng) was reverse-transcribed using the GoScript Reverse Transcription System (Promega) with the corresponding primers. RT-qPCR was performed with TransStart Green qPCR SuperMix (TransGen) on a Roche LightCycler 96 System. Data were normalized to the level of β-actin (mRNA) or U6 (microRNAs). Relative primers are shown in Table 3.

**Table 3:** Oligos and antibodies used in the research.

miR-155-5p-RT	GTCGTATCCAGTGCAGGGTCCGAGGTATTCGCACTGGATACGACACCCCT	For miR-155-5p RT-qPCR
miR-155-5p-F	GCGCTTAATGCTAATCGTGAT	
miR-155-5p-R	CCAGTGCAGGGTCCGAGGTA	
miR-574-5p-RT	GTCGTATCCAGTGCAGGGTCCGAGGTATTCGCACTGGATACGACACACAC	For miR-574-5p RT-qPCR
miR-574-5p-F	GGCTGAGTGTGTGTGTGTGA	
miR-574-5p-R	CCAGTGCAGGGTCCGAGGTA	
miR-187-3p-RT	GTCGTATCCAGTGCAGGGTCCGAGGTATTCGCACTGGATACGACCCGGCT	For miR-187-3p RT-qPCR
miR-187-3p-F	GGCTCGTGTCTTGTGTTGC	
miR-187-3p-R	CCAGTGCAGGGTCCGAGGTA	
miR-130a-3p-T	GTCGTATCCAGTGCAGGGTCCGAGGTATTCGCACTGGATACGACATGCCC	For miR-130a-3p RT-qPCR
miR-130a-3p-F	GCGCCAGTGCAATGTTAAAA	
miR-130a-3p-R	CCAGTGCAGGGTCCGAGGTA	
miR-181b-5p-RT	GTCGTATCCAGTGCAGGGTCCGAGGTATTCGCACTGGATACGACACCCAC	For miR-181b-5p RT-qPCR
miR-181b-5p-F	GCAACATTCATTGCTGTCG	
miR-181b-5p-R	CCAGTGCAGGGTCCGAGGTA	
miR-361-5p-RT	GTCGTATCCAGTGCAGGGTCCGAGGTATTCGCACTGGATACGACGTACCC	For miR-361-5p RT-qPCR
miR-361-5p-F	GCGCTTATCAGAATCTCCAG	
miR-361-5p-R	CCAGTGCAGGGTCCGAGGTA	
miR-16-RT	GTCGTATCCAGTGCAGGGTCCGAGGTATTCGCACTGGATACGACCGCCAA	For miR-16 RT-qPCR
miR-16-F	GCGCGCTAGCAGCACGTAAATA	
miR-16-R	CCAGTGCAGGGTCCGAGGTA	
GAPDH-F	GCACCGTCAAGGCTGAGAAC	For GAPDH RT-qPCR
GAPDH-R	TGGTGAAGACGCCAGTGGA	
U6-F	CTCGCTTCGGCAGCACA	For U6 RT-qPCR
U6-R	AACGCTTCACGAATTTGCGT	
ACOT12-F	TGCTGGAGTTTCCTGCGTTAC	For ACOT12 RT-qPCR
ACOT12-R	GCATATCCTGTACCATGACCTTG	
TWIST2-F	ACAGCAGTGACATCGGACAG	For TWIST2 RT-qPCR
TWIST2-R	CCCCAAACATAAGACCCAGA	
Vimentin-F	GAGAACTTTGCCGTTGAAGC	For Vimentin RT-qPCR
Vimentin-R	GCTTCCTGTAGGTGGCAATC	
N-cadherin-F	ACAGTGGCCACCTACAAAGG	For N-cadherin RT-qPCR
N-cadherin-R	CCGAGATGGGGTTGATAATG	

### Western blotting

Exosomes or cells were lysed using RIPA buffer containing 1 mM PMSF (Beyotime). Lysates were separated on 12% SDS-PAGE gels (BBI) and transferred to PVDF membranes (Millipore). After blocking in 5% nonfat milk for 2 h, the membranes were processed according to an enhanced chemiluminescence (ECL) western blotting protocol (GE Healthcare) and scanned with Amersham Imager 680 (GE Healthcare). The following antibodies were used: anti-CD63 (Abcam, Ab213090); anti-CD9 (Abcam, Ab92726); anti-GM130 (Abcam, Ab52649); anti-ACOT12 (Thermo, MA5-25428); anti-TWIST2 (Proteintech, 11752-1-AP); anti-vimentin (Proteintech, 10366-1-AP); anti-N-cadherin (Proteintech, 22018-1-AP); and anti-β-actin (Sangon, D110001). Antibody validation data are provided on the manufacturers’ websites. Original WB images are shown in “Supplementary Material”.

### Immunohistochemistry (IHC)

Resected tumor tissues were fixed in 4% paraformaldehyde, embedded in paraffin and cut into 4-μm sections. Then, the sections were treated with xylene and ethanol to remove paraffin. After blocking with 5% normal goat serum, the slices were incubated with anti-ACOT12 (Thermo, MA5-25428), anti-TWIST2 (Proteintech, 11752-1-AP), anti-vimentin (Proteintech, 10366-1-AP), anti-N-cadherin (Proteintech, 22018-1-AP) or anti-Ki67 (Proteintech, 27309-1-AP) overnight at 4 °C and washed with PBS 3 times. Subsequently, the slices were incubated with the HRP-conjugated secondary antibody and streptavidin–peroxidase. ImageJ software was used to measure the average integral optical density of each positively stained slide. Three random fields were selected from each section for measurement.

### Dual-luciferase reported assay

The biological prediction database TargetScan (http://www.targetscan.org) was used to predict the potential binding sites of miR-155-5p in the ACOT12 sequence, and the results were verified with dual-luciferase assays. Briefly, ACOT12 fragments containing the predicted wild-type (WT) or mutant (MUT) binding sites were synthesized and cloned into pmiR-RB-ReportTM (RiboBio). 293 T cells were transfected with 200 ng luciferase reporter plasmid containing either WT or MUT 3’ UTR of ACOT12 by Lipofectamine 3000 reagent (Invitrogen). Relative luciferase activity was measured with a dual-luciferase reporter assay system (Promega, E1910) after 48 h of transfection. Firefly luciferase activity was normalized to the corresponding Renilla luciferase activity.

### Tumor xenograft experiments

BALB/c nude mice (male, aged 4–5 weeks, 18–20 g) obtained from Yangtze Delta Region Institute of Tsinghua University (Hangzhou, Zhejiang, China) were included in this study. To establish xenografts, U87 cells (1 × 10^7^ cells in 200 μL PBS) were subcutaneously injected into the flanks of nude mice. After 7 days, the transplanted nude mice were randomly divided into two groups (mice without tumor formation were excluded). MiR-155-5p antagomir or NC (RiboBio) was directly injected into the implanted tumor at a dose of 10 nmol (in 100 μL PBS) every 3 days 7 times (double blindly design, antagomir or NC were blindly injected). Tumor volume (V) was monitored by measuring the length (*L*) and width (*W*) every 3 days and calculated with the formula: *V* = 0.5 × length × width^2^. The mice were sacrificed after 28 days, and the tumor tissues were collected for subsequent analysis. All animal studies were approved by the Institutional Animal Care and Use Committee of the First Affiliated Hospital of USTC.

### Statistical analysis

All data were analyzed with SPSS (version 23.0) or GraphPad Prism software (version 7.0) and expressed as the mean ± SD from three independent experiments. All experiments in this work were replicated at least three times. The differences between two groups were analyzed by Student’s *t*-test, and one-way analysis of variance (ANOVA) was used for multiple groups. The Kaplan–Meier method was used to assess the overall survival rate of patients. Differences with *P* < 0.05 were considered statistically significant.

## Supplementary information

Supplementary information

Supplemental Material

checklist

## Data Availability

The data generated or analyzed during this study are included in this article and its additional information files.
