# Supplementary material for: Exosomal miR-155-5p derived from glioma stem-like cells promotes mesenchymal transition via targeting ACOT12
Source: Cell Death Dis. 2022 Aug 19;13(8):725. doi: 10.1038/s41419-022-05097-w (PMC9391432; doi:10.1038/s41419-022-05097-w)
Supplement: Supplementary file 1 — Supplementary information [file 41419_2022_5097_MOESM1_ESM.docx]

**Inventory of supplementary information**

**1.Fig S1-S5**


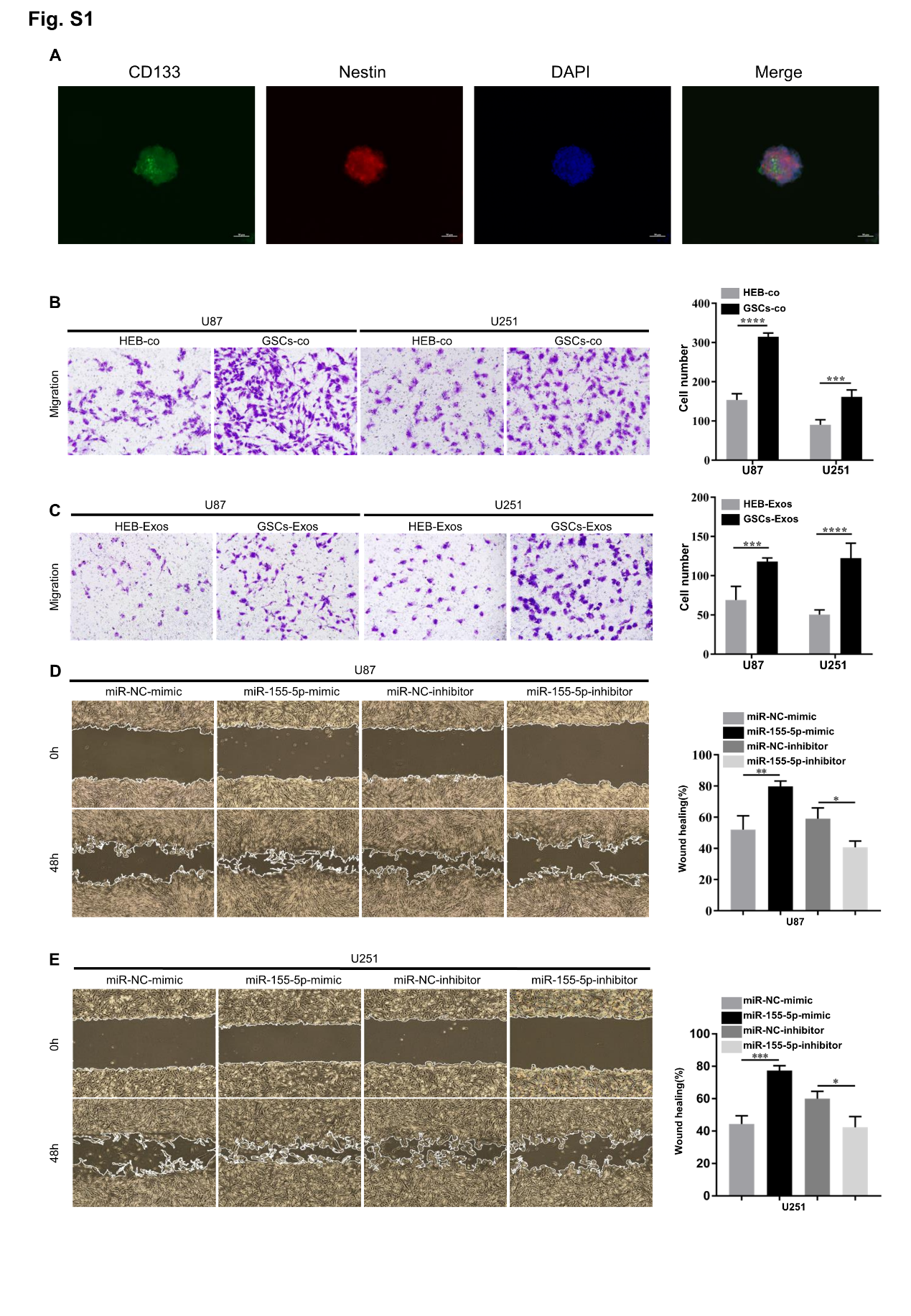


**Fig. S1 Characterization of glioma stem-like cells and the effect of GSCs, GSCs-derived exosomes and miR-155-5p on the migration of glioma cells**

**A** Immunofluorescence images of the GSC markers CD133 and Nestin. Scale bar, 50 μm; **B** The migration of glioma cells after coculture with GSCs or HEB cells was assessed using Transwell assays without Matrigel; **C** The migration of glioma cells incubated with exosomes secreted by GSCs or HEB cells was assessed using Transwell assays without Matrigel; **D** Wound healing assays of glioma cells with overexpression or inhibition of miR-155-5p. Representative images are shown. Data represent as the mean ± SEM (repetition=3). ***P < 0.001; ****P < 0.0001 (Student’s t test).


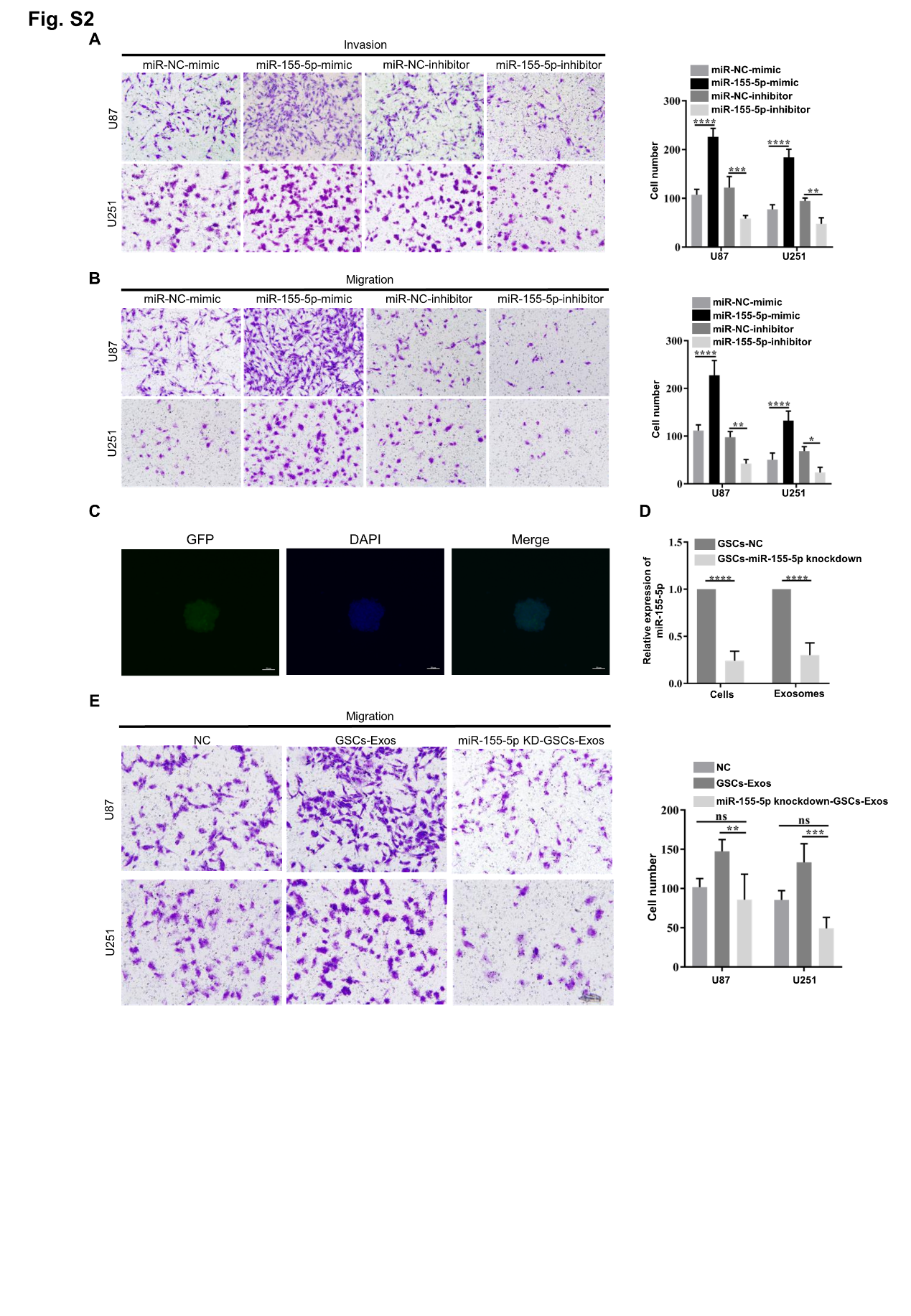


**Fig. S2 The role of exosomal miR-155-5p in glioma invasion and migration**

**A** Wound healing assays of glioma cells with overexpression or inhibition of miR-155-5p; **B** The invasion of glioma cells with overexpression or inhibition of miR-155-5p, as measured by Matrigel-coated Transwell assays; **C** Immunofluorescence images of the GSC transfected with miR-155-5p knockdown GFP lentivirus. Scale bar, 50 μm; **D** The level of miR-155-5p in GSCs and GSCs-derived exosomes was detected by RT-qPCR; **E** The migration of glioma cells treated with GSCs-derived exosomes or miR-155-5p knockdown GSCs-derived exosomes, as measured by Transwell assays without Matrigel. Representative images are shown. Data represent as the mean ± SEM (repetition=3); ns=not statistically significant; *P < 0.05; **P < 0.01; ***P < 0.001; ****P < 0.0001 (Student’s t test).


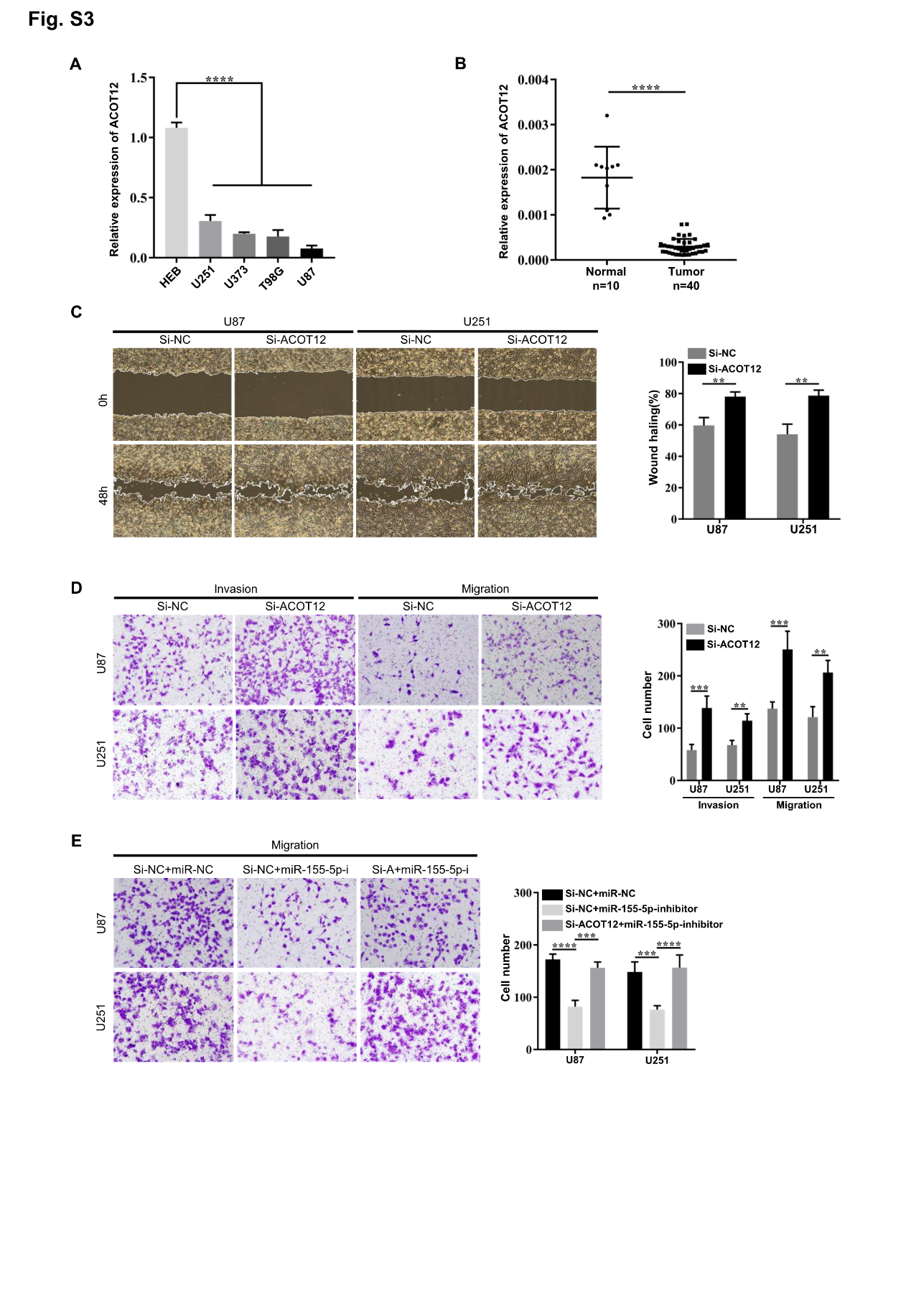


**Fig. S3 The expression level and function of ACOT12 in glioma**

**A** Relative expression level of ACOT12 in HEB cells and four glioma cell lines detected by RT-qPCR. **B** Relative expression of ACOT12 in 10 normal brain tissues and 40 glioma tissues detected by RT-qPCR. **C, D** The effect of ACOT12 on the migration and invasion of glioma cells measured by wound healing assays and Transwell assays. **E** The migration of glioma cells transfected with miR-155-5p inhibitor alone or cotransfected with si-ACOT12 and miR-155-5p inhibitor. Representative images are shown. Data represent as the mean ± SEM (repetition=3); ns=not statistically significant; **P < 0.01; ***P < 0.001; ****P < 0.0001 (Student’s t test).


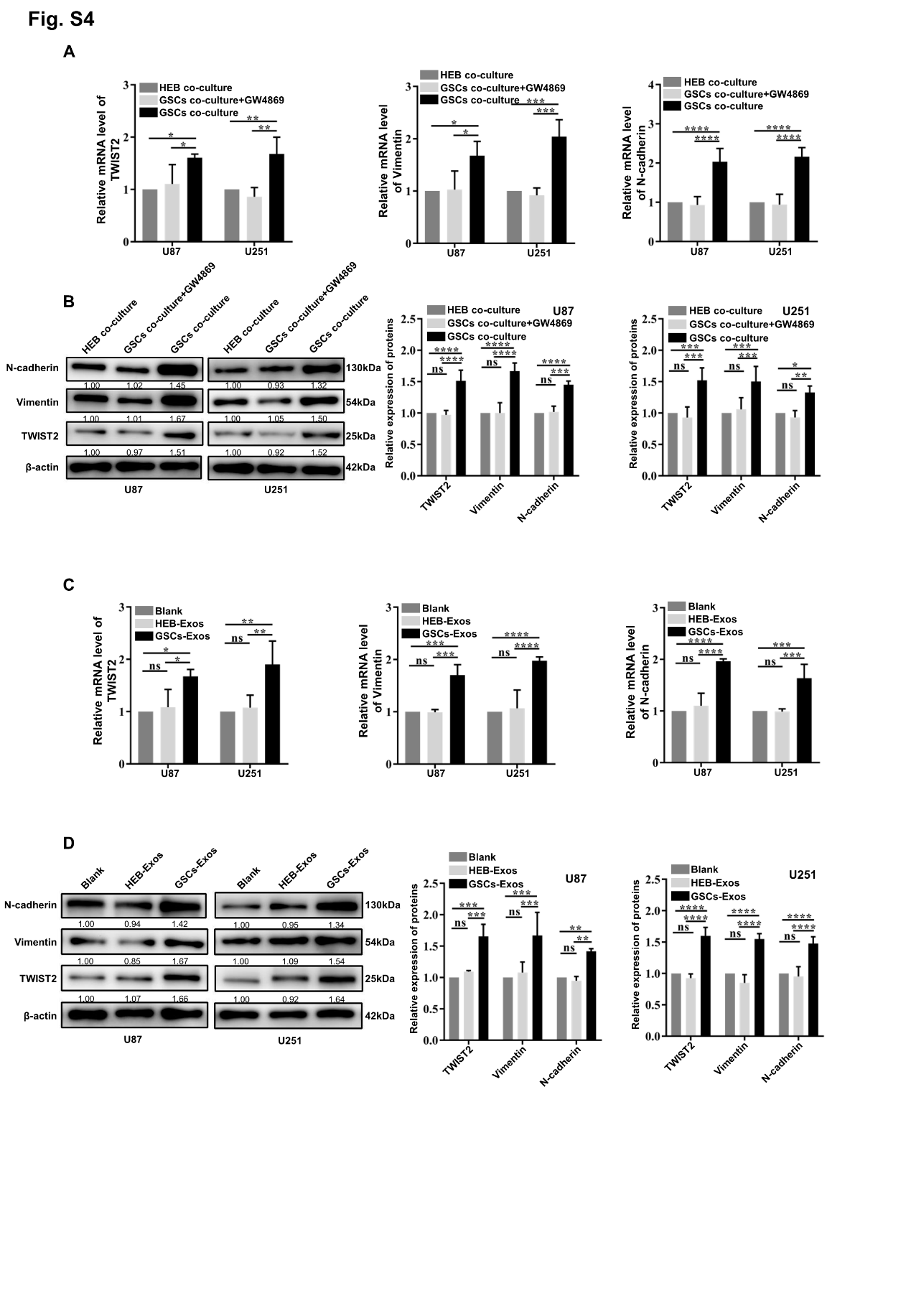


**Fig. S4 Both GSCs and GSCs-derived exosomes induce mesenchymal transition in glioma cells**

**A, B** Relative mRNA and protein levels of mesenchymal transition markers in glioma cells after coculture with GSCs or GSCs treated with GW4869; **C, D** Relative mRNA and protein levels of mesenchymal transition markers in glioma cells incubated with exosomes secreted by GSCs or HEB cells. Data represent as the mean ± SEM (repetition=3); ns=not statistically significant; *P < 0.05; **P < 0.01; ***P < 0.001; ****P < 0.0001 (Student’s t test).


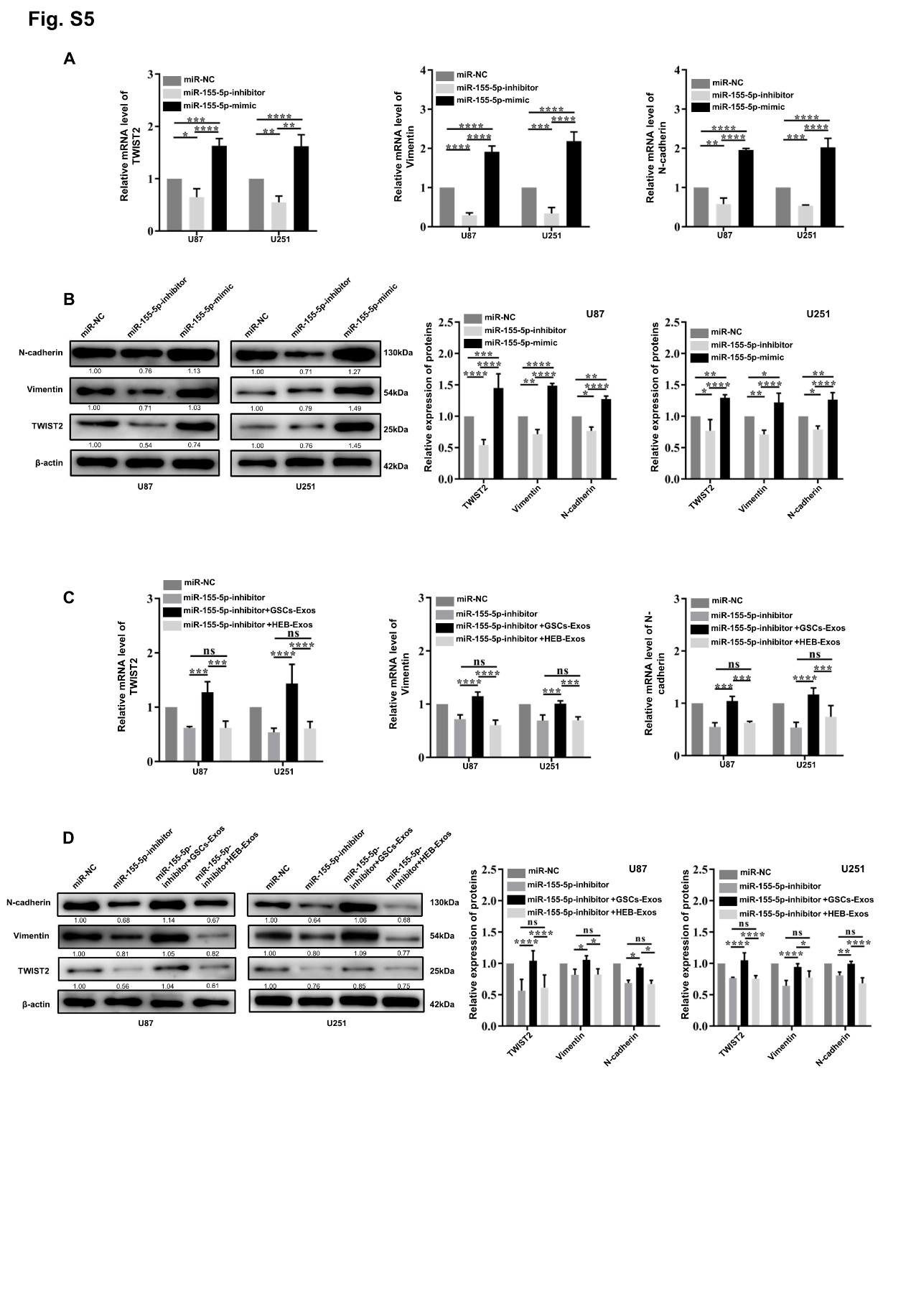


**Fig. S5 GSCs-derived exosomes can restore the influence of miR-155-5p on mesenchymal transition**

**A, B** Relative mRNA and protein levels of mesenchymal transition markers in glioma cells transfected with miR-155-5p mimic or inhibitor; **C, D** Relative mRNA and protein levels of mesenchymal transition markers in glioma cells treated with miR-155-5p inhibitor and exosomes secreted by GSCs or HEB cells. Data represent as the mean ± SEM (repetition=3); ns=not statistically significant; *P < 0.05; **P < 0.01; ***P < 0.001; ****P < 0.0001 (Student’s t test).
