# Supplementary figures and images for: Exosomal miR-155-5p derived from glioma stem-like cells promotes mesenchymal transition via targeting ACOT12
Source: Cell Death Dis. 2022 Aug 19;13(8):725. doi: 10.1038/s41419-022-05097-w (PMC9391432; doi:10.1038/s41419-022-05097-w)

# Original Western Blots

Fig. 1D

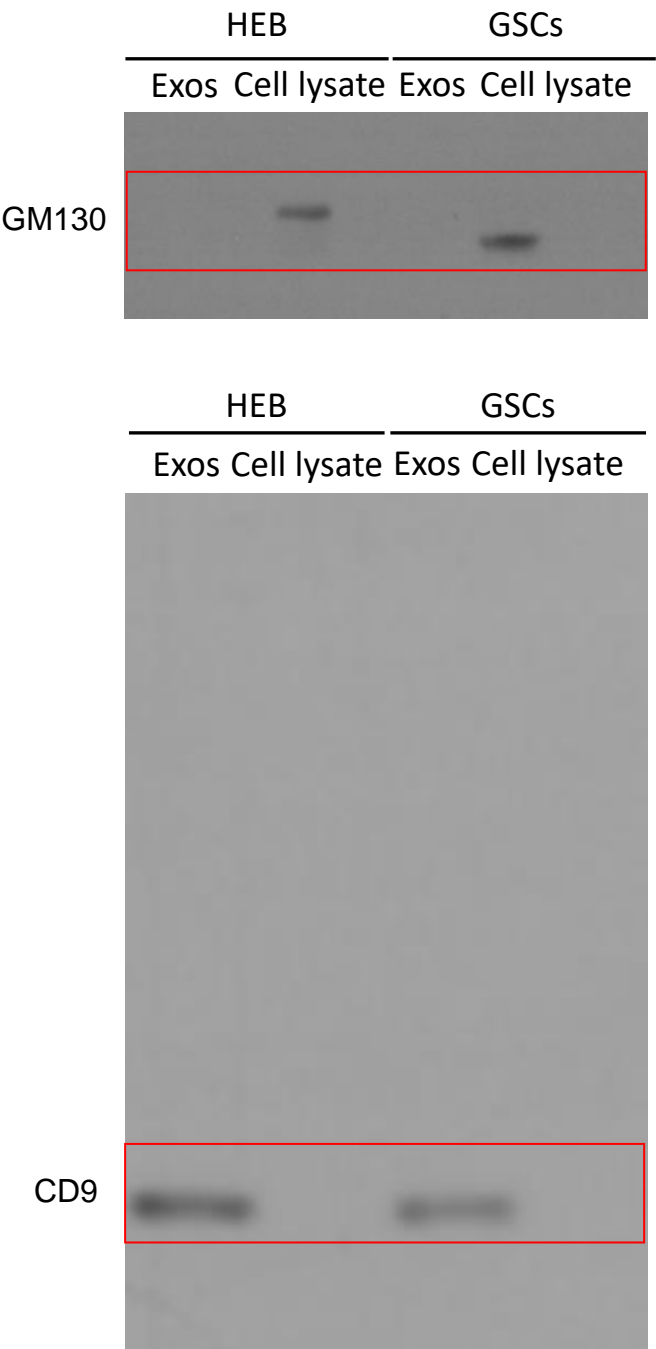

Fig. 1D

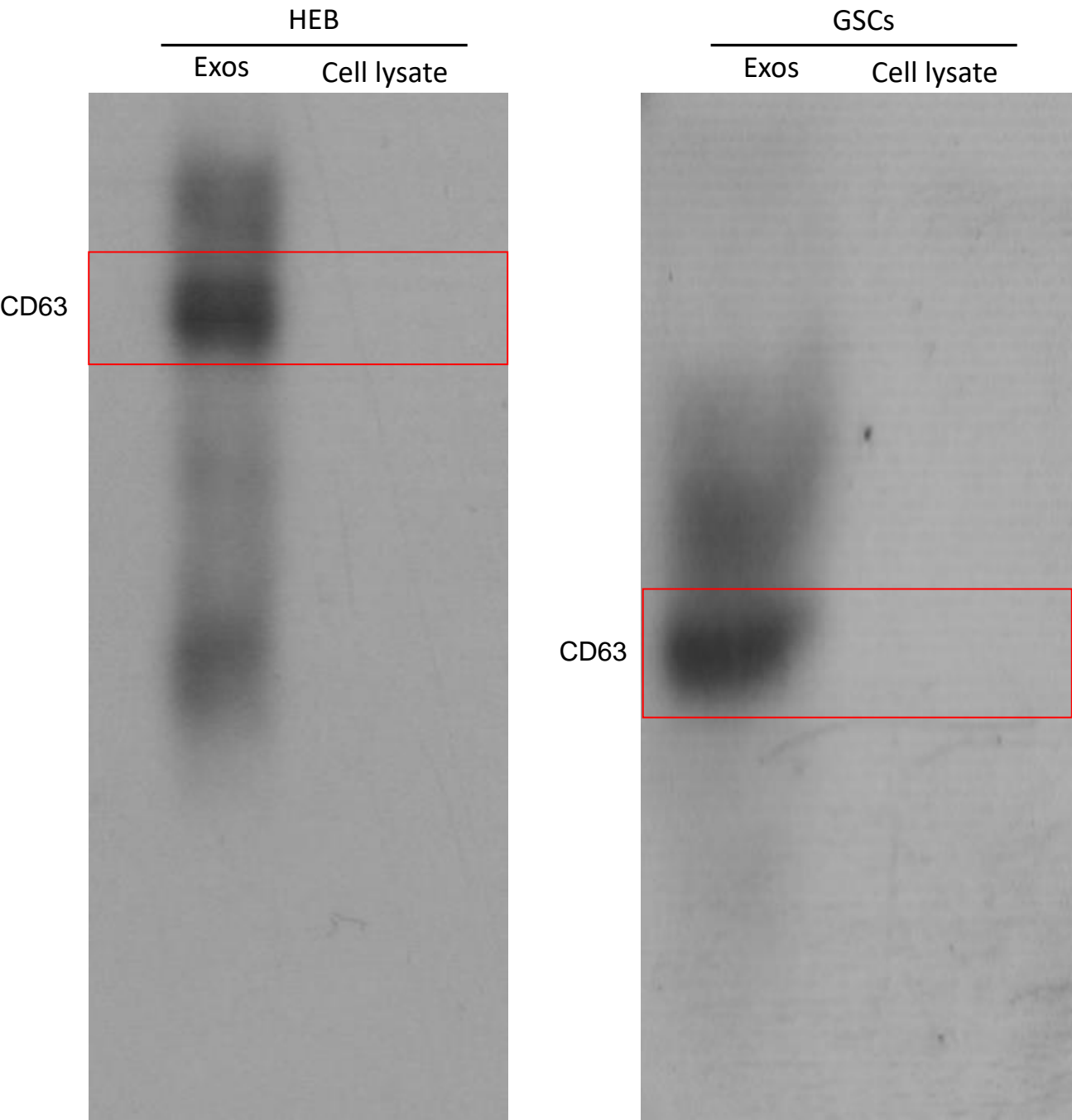

Fig. 5D

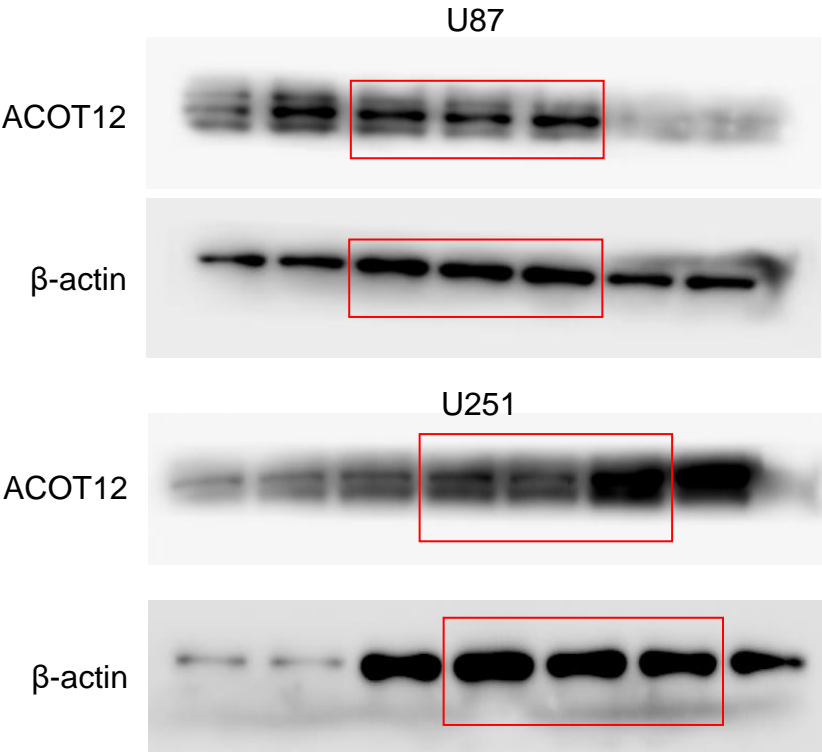

Fig. 5F

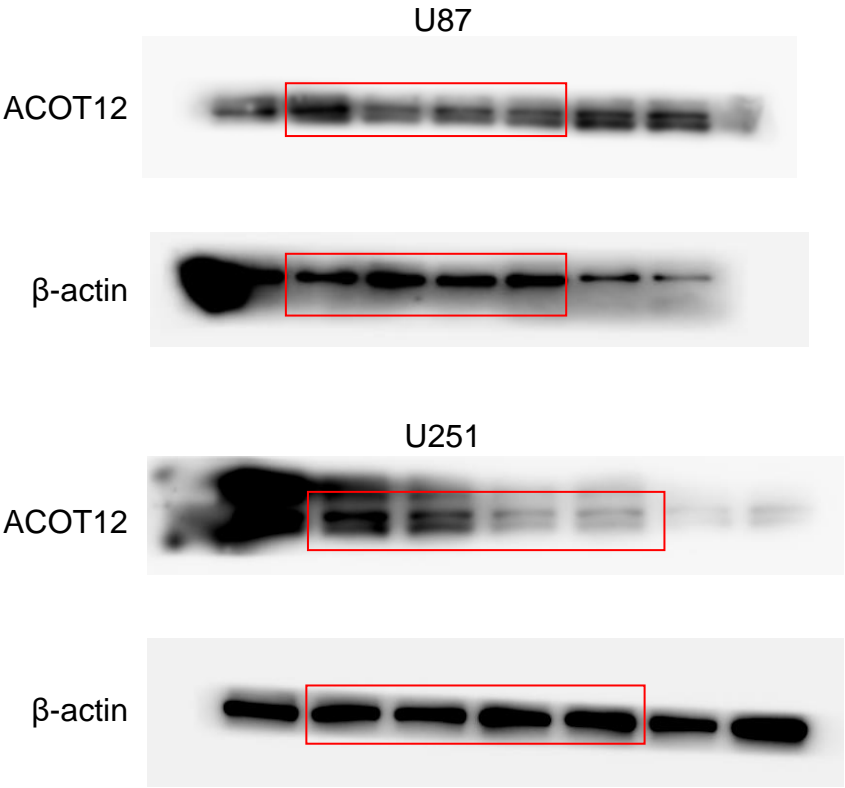

Fig. 6B

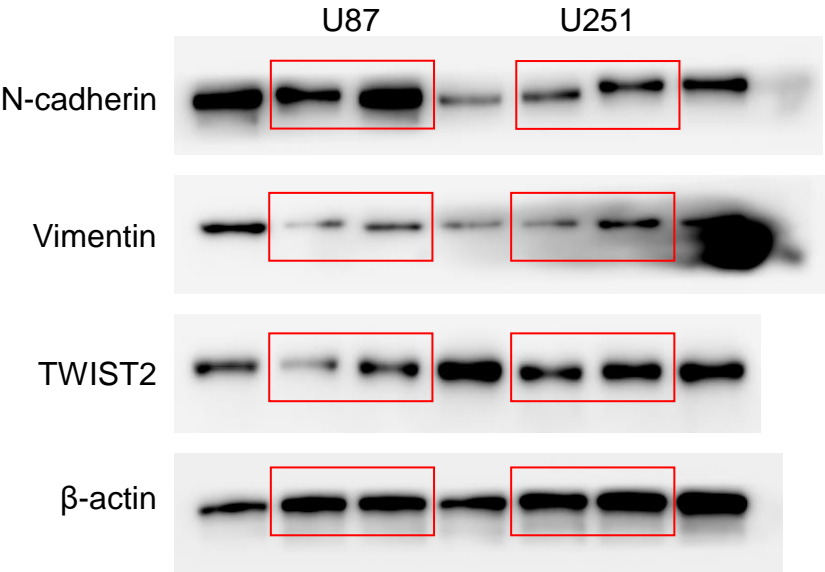

Fig. 6D

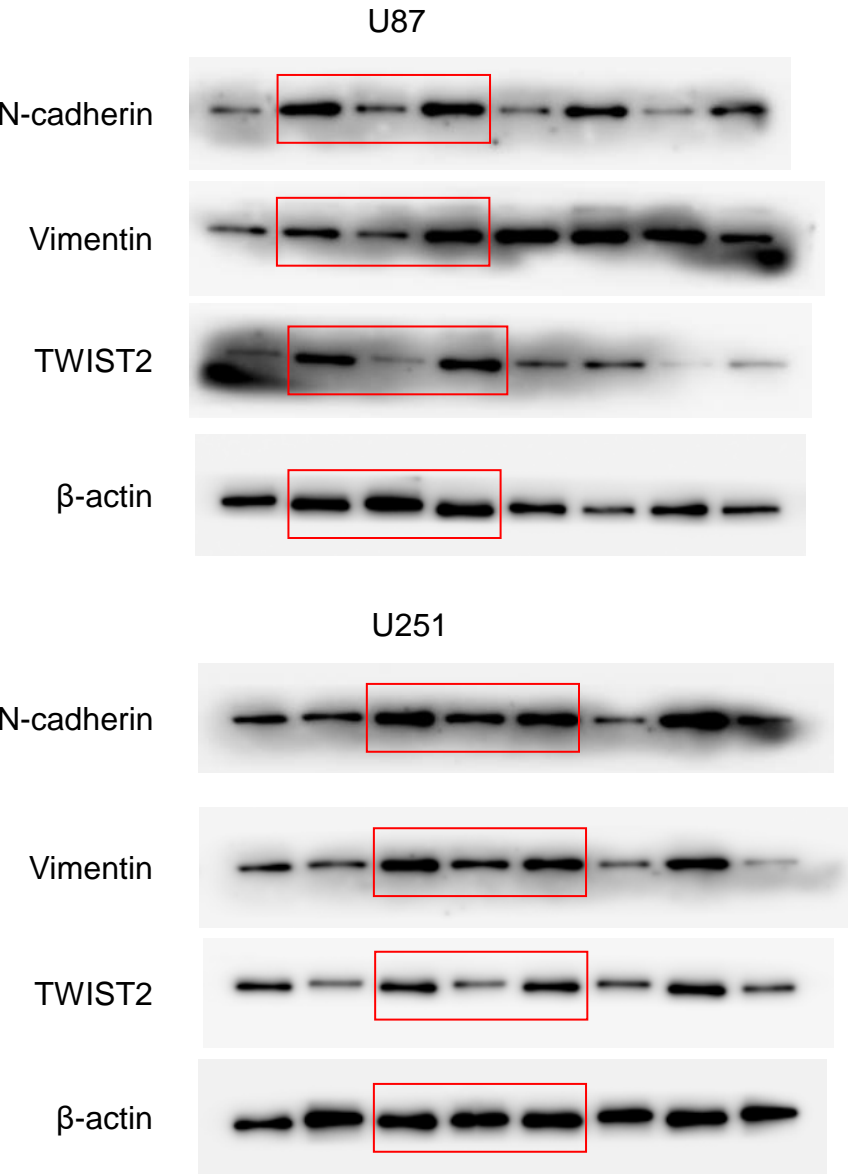

Fig. S4B

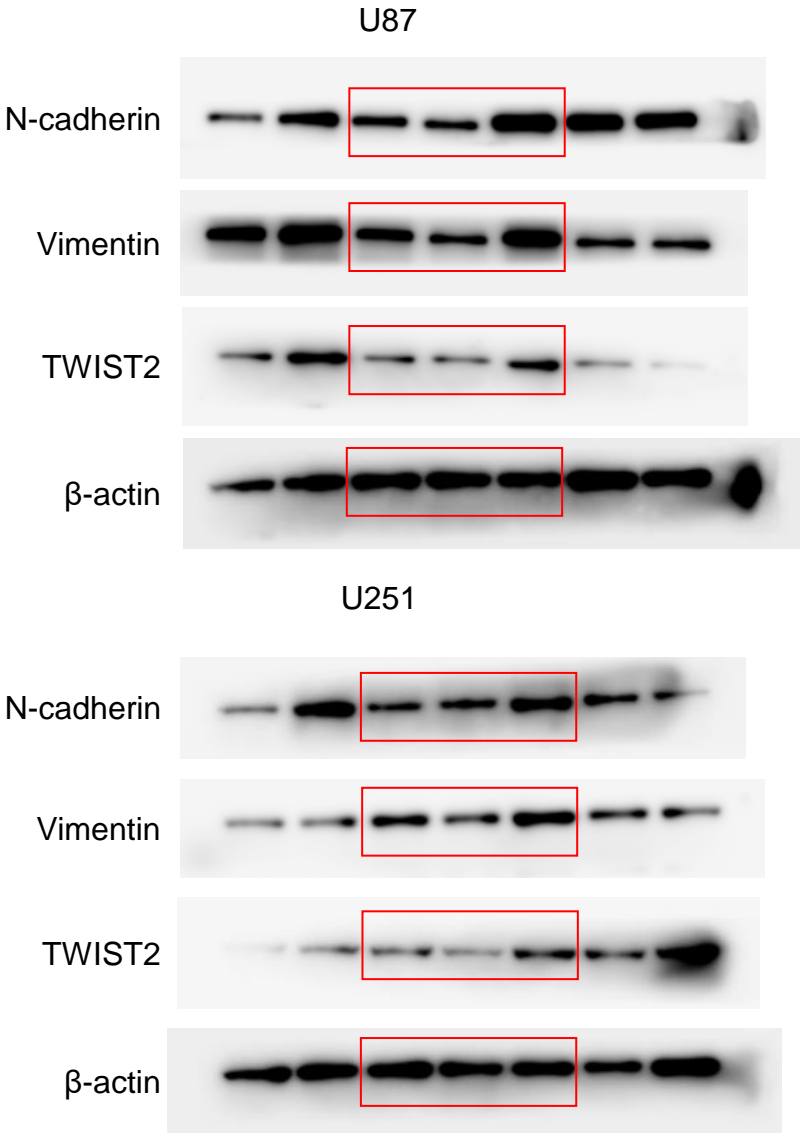

Fig. S4D

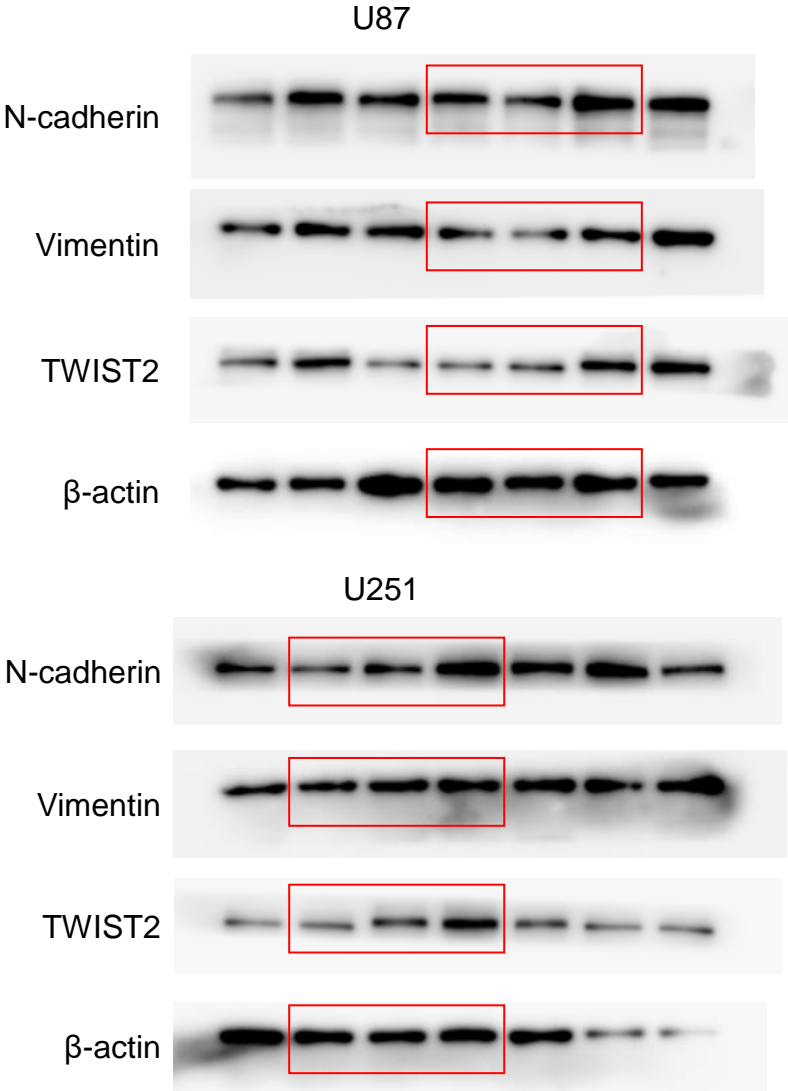

Fig. S5B

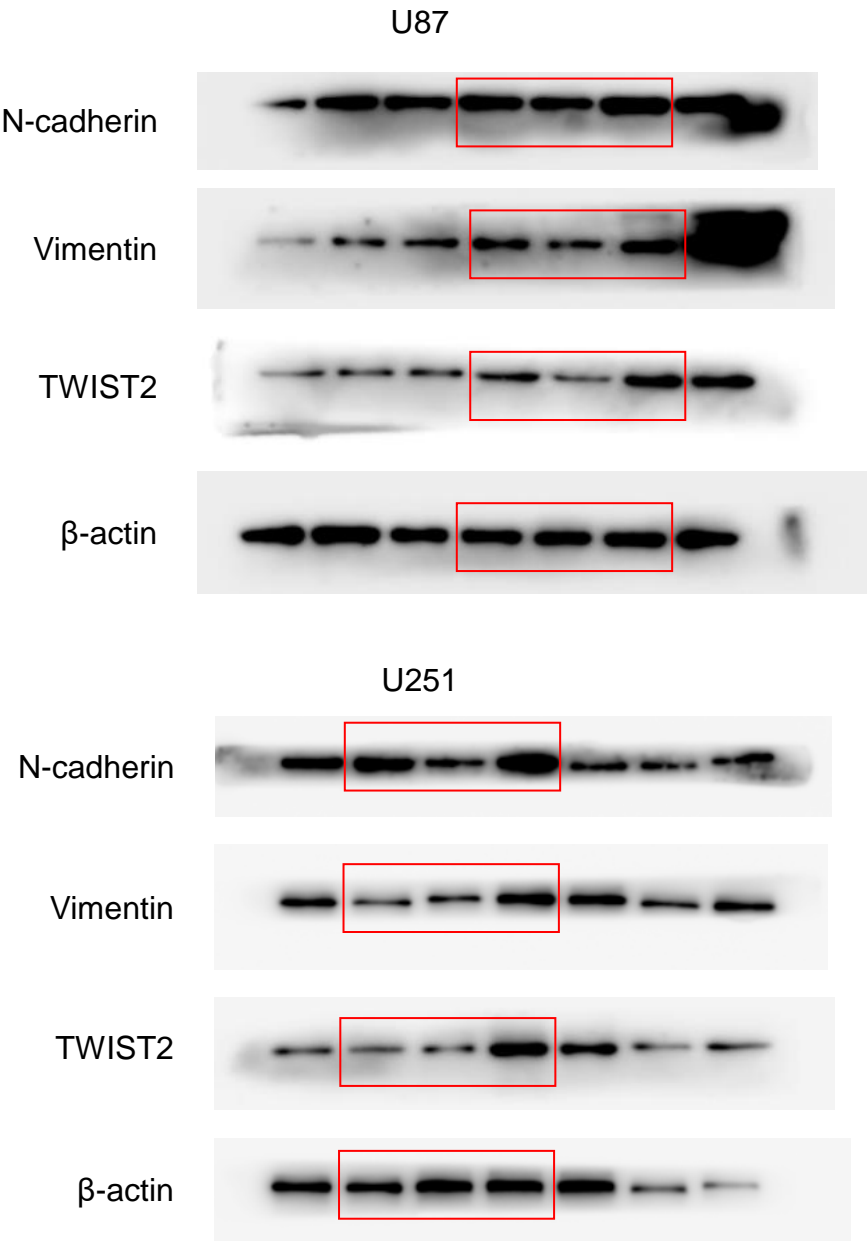

Fig. S5D

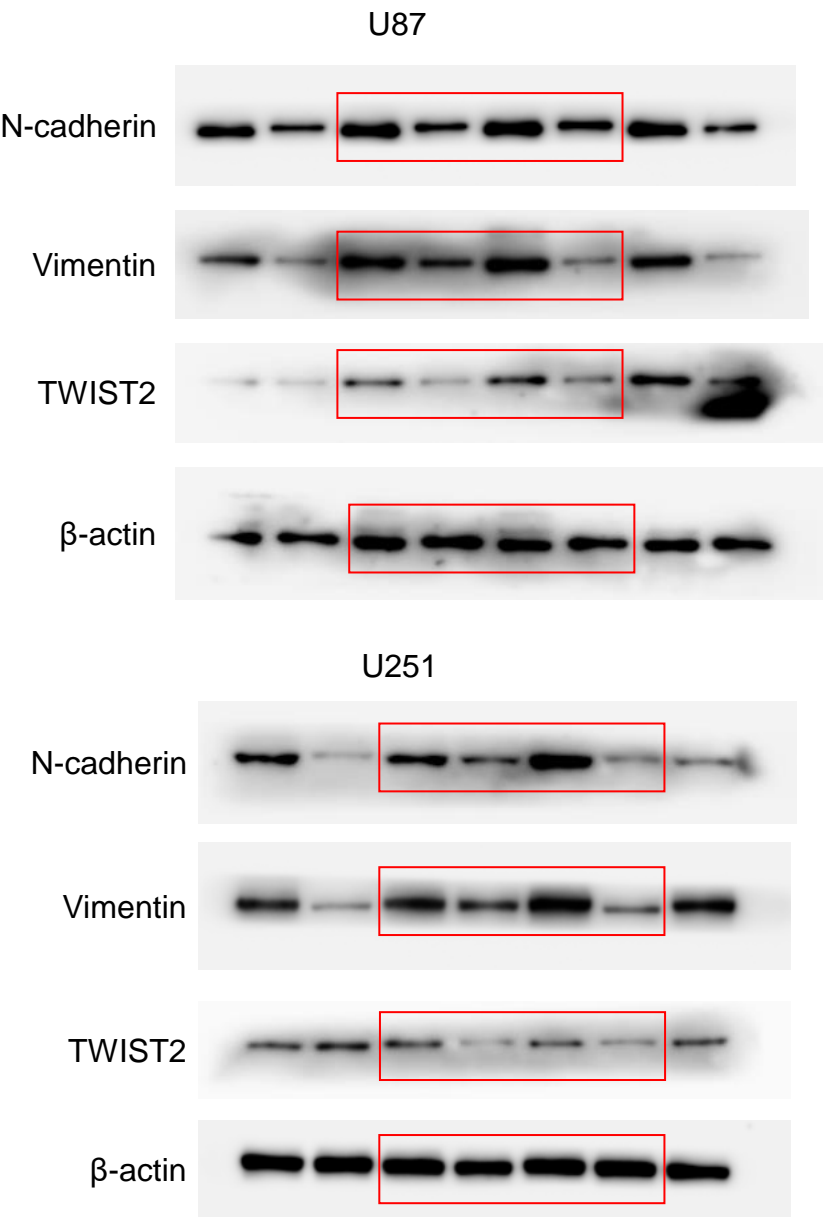

Supplement: Supplementary file 2 — Supplemental Material [file 41419_2022_5097_MOESM2_ESM.pdf]
